# Functional maps of a genomic locus reveal confinement of an enhancer by its target gene

**DOI:** 10.1126/science.ads6552

**Published:** 2025-09-18

**Authors:** Mathias Eder, Christina J.I. Moene, Lise Dauban, Mikhail Magnitov, Jamie Drayton, Marcel de Haas, Christ Leemans, Martijn Verkuilen, Elzo de Wit, Anders S. Hansen, Bas van Steensel

**Affiliations:** 1 Division of Gene Regulation and Netherlands Cancer Institute; Amsterdam, the Netherlands; 2 Division of Molecular Genetics, Netherlands Cancer Institute; Amsterdam, the Netherlands; 3 Oncode Institute; the Netherlands; 4 Department of Biological Engineering, Massachusetts Institute of Technology; Cambridge, MA 02139, USA; 5 The Broad Institute of MIT and Harvard; Cambridge, MA 02139, USA; 6 Koch Institute for Integrative Cancer Research; Cambridge, MA, 02139, USA; 7 The Novo Nordisk Foundation Center for Genomic Mechanisms of Disease, Broad Institute of MIT and Harvard, Cambridge, MA 02142, USA

## Abstract

Genes are often activated by enhancers located at large genomic distances. The importance of this positioning is poorly understood. By relocating promoter-reporter constructs into thousands of alternative positions within a single locus, we dissected the positional relationship between the mouse *Sox2* gene and its distal enhancer. This revealed an intricate, sharply confined activation landscape, in which the native *Sox2* gene occupies an optimal position for its activation. Deletion of the gene relaxes this confinement and broadly increases reporter activity. The confining effect of the *Sox2* gene is partially conferred by its ~1 kb coding region. Our local relocation approach provides high-resolution functional maps of a genomic locus and reveals that a gene can strongly constrain the realm of influence of its enhancer.

Mammalian enhancers can activate genes across large genomic distances, up to hundreds of kilobases (1, 2), presumably by contacting their target promoter through folding of the chromatin fiber. Indeed, enhancers tend to have increased contact with the promoters they regulate (3), but it is unclear whether this is a cause or effect of their function. In addition, enhancer – promoter pairs often reside within the same topologically associated domain (TAD), a region of preferential self-interaction in the genome that is often demarcated by CTCF binding sites (4, 5). These TADs are thought to form functional domains (6, 7), but it is largely unclear whether enhancers can activate promoters anywhere within a TAD, or whether some positions within a TAD are more favorable than others. Recent studies in two loci with near-random three-dimensional (3D) structure indicated that promoter activity increases with decreasing genomic distance to its enhancer (8, 9). However, in more structured genomic loci the effect of position on enhancer – promoter communication has remained elusive. Here, we addressed these questions by constructing high-resolution maps of promoter activity as function of position in a native locus with pronounced 3D folding.

## High throughput relocation by Sleeping Beauty transposition

**Fig. 1:**
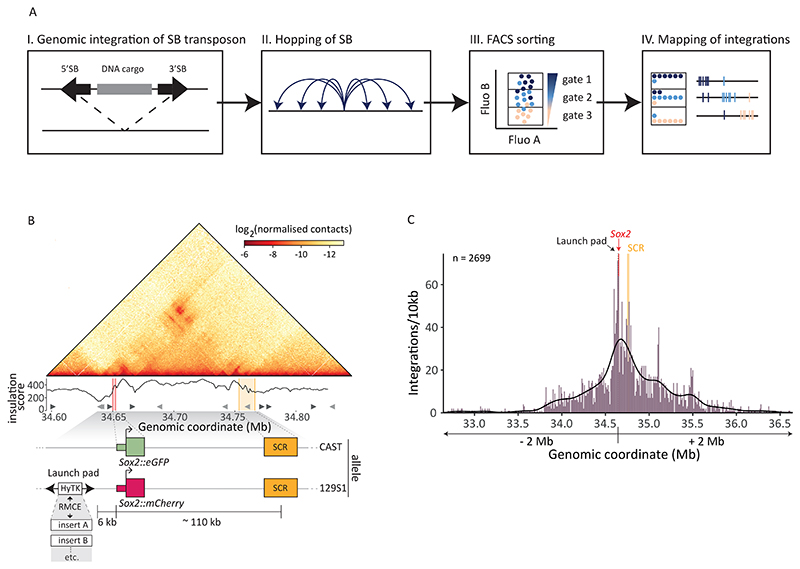
Sleeping Beauty mobilization creates integrations covering the *Sox2* locus. **(A)** Workflow of systematic SB-relocation. **I**. Integration of SB transposon into locus of interest by Cas9 editing; **II**. Cut-and-paste relocation of Sleeping Beauty (SB) transposon by transient expression of SB transposase; **III**. Enrichment of cells with desired phenotype by fluorescence-activated cell sorting (FACS); **IV**. Mapping of integrations from sorted cell pools to link expression to location. **(B)** Overview of the *Sox2* locus. Top to bottom: RCMC contact map in mESCs (18) (1 kb resolution) and derived insulation scores; positions and orientations of CTCF binding sites (triangles); positions of SCR and eGFP/mCherry tagged *Sox2* alleles, the SCR, and a launch pad (129S1 allele) containing SB with a recombination cassette. **(C)** Distribution of hopped SB carrying a 282 bp inert DNA sequence, mobilized from the launch pad; *n* is total number of integrations mapped in the plotted region; one biological replicate.

To unravel the logic that underlies the linear arrangement of regulatory elements, it is necessary to alter the genomic position of such elements systematically and probe the functional consequences. Here we provide such a systematic transplantation of regulatory elements by adapting a methodology based on the cut-and-paste mechanism of transposable elements (9–11). When mobilized from the genome by its transposase, the Sleeping Beauty (SB) transposon excises and re-integrates, usually within ~1-2 Mb from the original location (10, 12). Thus, any sequence inserted between the two SB inverted terminal repeats (ITRs) can be randomly relocated (“hopped”) throughout a genomic locus. We developed a workflow to generate and map thousands of integrations in a single locus, and link the locations to the expression of a transcribed reporter, yielding high-resolution functional maps of the local regulatory landscape (Fig. 1A).

For this study we chose the *Sox2* locus in mouse embryonic stem cells (mESCs). In these cells, *Sox2* expression is primarily determined by the *Sox2* control region (SCR), which is a cluster of enhancers located 110 kb downstream of the gene in the same TAD (13–17). Within this TAD, the *Sox2* gene has been found to preferentially contact the SCR (18) (Fig. 1B). The 1.5 Mb region around *Sox2* contains no other protein-coding genes (13). To ease genetic engineering, we used F1-hybrid (*129/Sv:CAST/EiJ*) mESCs, in which the *CAST* and the *129S1* alleles can be distinguished by a high density of sequence polymorphisms. We tagged the two alleles of *Sox2* with *eGFP* (*CAST*) and *mCherry* (*129S1*) to be able to quantify allelic *Sox2* expression by flow cytometry (Fig. S1A). To confirm the regulation by the SCR, we transfected the dual-tagged cell line with Cas9 and two gRNAs that cause deletion of the SCR. In line with the reported key role of the SCR (13, 14, 16), a proportion of cells lost more than 90% of *eGFP* or *mCherry* expression, most likely due to a deletion of the SCR on the corresponding allele (Fig. S1B).

Next, we used Cas9 editing to create a “launch pad” by integrating a SB transposon cassette 6 kb upstream of the *Sox2::mCherry* allele (-116 kb relative to the SCR, Fig. 1B). The cassette contained a double selection marker (mPGK-HyTK) flanked by a pair of heterotypic Flp-recombinase recognition sites (FRT/F3), enabling us to easily change the DNA sequence within the transposon by recombination-mediated cassette exchange (RMCE) (Fig. S1C). In a proof-of-principle experiment, we induced hopping of the SB cassette containing an arbitrary 282 bp sequence (see methods). After culturing the cells for one week to dilute the transposase-containing plasmid and prevent further re-hopping events, we isolated and expanded 200,000 cells and mapped the integrations using a Tn5-based sequencing approach (19). In some of the cells, the transposon was never mobilized, which is reflected by 33% of the mapping reads originating from the launch site. Of all the hopped integrations, 72% were located on the same chromosome as the *Sox2* locus (chr3) (Fig. S1D) and 97% of the integrations that could be mapped allele-specifically were found in *cis* (Fig. S1E). Within -/+ 2 Mb of the launch pad we mapped 2699 unique integrations (Fig. 1C), which were equally distributed between the forward (1342) and reverse (1357) orientations (Fig. S1F). We note that this mapping is not exhaustive, and the actual pool likely contains more integrations, since more than 70% of the integrations were only supported by one unique mapping read (Fig. S1G). This experiment demonstrates the effectiveness of SB hopping to generate thousands of genomic integrations.

## A highly detailed activation landscape of the *Sox2* locus

**Fig. 2:**
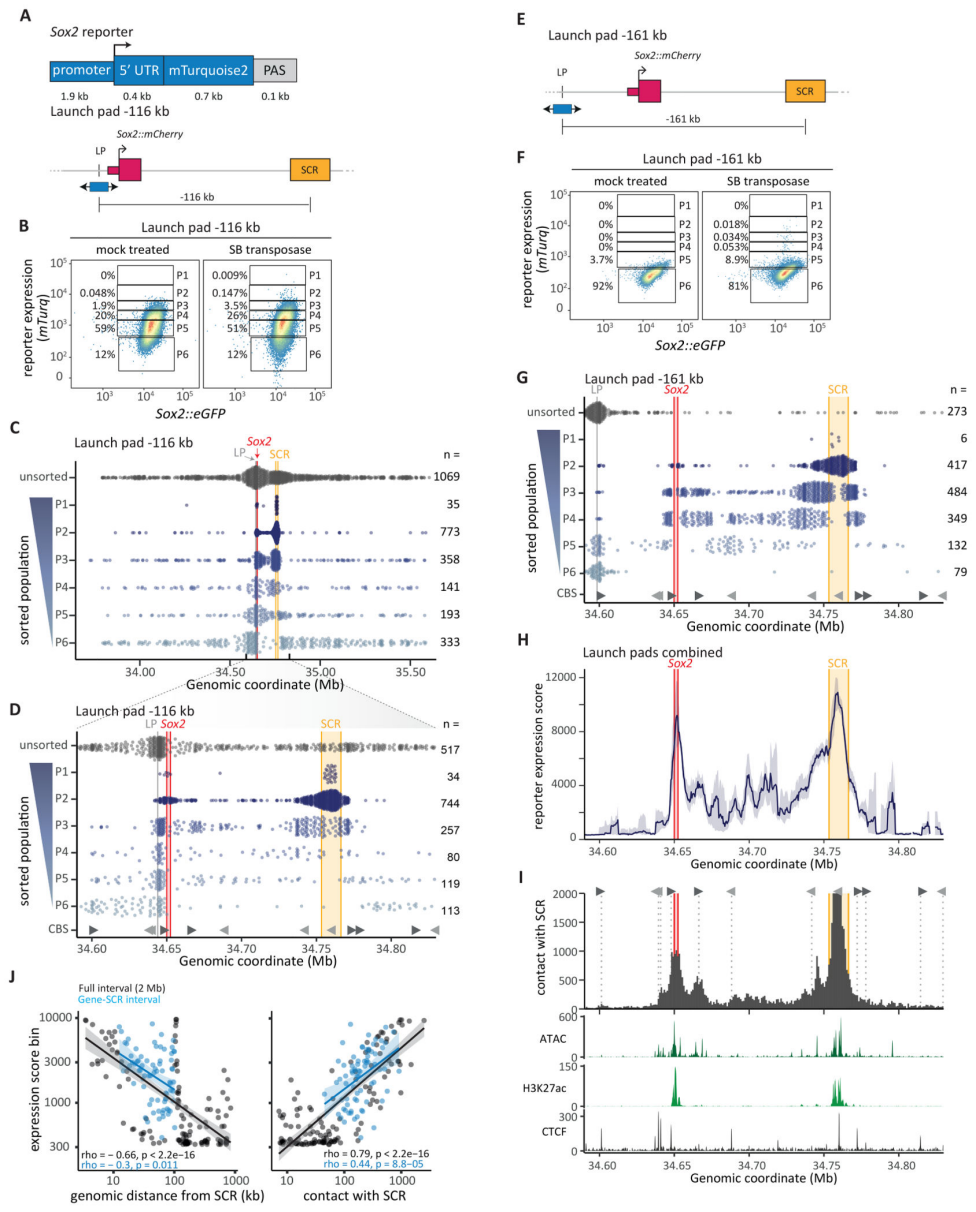
A highly detailed activation landscape of the *Sox2* locus. **(A)** Top panel: design of mTurquoise2 reporter driven by *Sox2* promoter and 5’UTR, followed by SV40 polyadenylation signal (PAS). Bottom panel: reporter was inserted in the -116 kb launch pad (LP), upstream of *Sox2::mCherry*. **(B)** Expression levels of reporter (mTurq) and *Sox2::eGFP* (control) in mock-transfected cells and in cells transfected with SB transposase, measured by FACS. Boxes mark sorting gates used to obtain cell populations P1-P6 in (C-D). The percentage of cells in each gate is indicated. Representative result of four biological replicates. **(C)** Mapped integrations from a large pool of unsorted cells and the six sorted populations (P1-P6), mobilized from the -116 kb launch pad. Each dot indicates one mapped integration, *n* indicates number of plotted integrations. Combined data from four biological replicates (see also Fig. S2C). LP, launch pad position. **(D)** Zoom-in of (C). Grey triangles indicate CBS positions and orientations. **(E-G)** Same as (A-B, D), except that the reporter was mobilized from a launch pad at position - 161 kb. Data are from 2 biological replicates combined. **(H)** Expression score derived from data shown in (D, G**)** and Fig. S2F combined, smoothened using a 5 kb window shifted in 500 bp steps and only plotted when the window contains 3 or more SB integrations. Shaded region indicates 95% confidence interval. **(I)** Top: virtual 4C contact profile (from RCMC data, (18)) with the center of the SCR as viewpoint, at 1 kb resolution. Dashed lines indicate CBSs and grey triangles indicate their orientation. Bottom: ATAC-seq signal (20), H3K27ac ChIP-seq p-value signal (21) and CTCF ChIP-seq coverage (our own data), smoothened over 500 bp. **(J)** Correlation between expression score (based on the three launch pads combined) and genomic distance to the SCR (left) or virtual 4C contact with the core SCR (right), per 1 kb bin on the region displayed in (C) (full interval in black) and the *Sox2*-SCR interval (from 5 kb after the 3’ end of *Sox2* to 5kb before the SCR) in blue. Only bins with 3 or more SB integrations are included. Rho is Spearman’s correlation coefficient.

Next, we set out to generate a detailed map of the expression potential of the *Sox2* locus, *i.e*., to determine quantitatively how the location throughout the locus affects the ability of the *Sox2* promoter to be activated. For this we engineered a reporter comprising 1.9 kb of the *Sox2* promoter (Sox2P, excluding a promoter-proximal CTCF binding site [CBS]) driving the expression of mTurquoise2 (*mTurq*), a blue fluorescent protein (Fig. 2A). We integrated this reporter into the launch pad using RMCE. This resulted in low but detectable reporter expression, at 9.7% of the expression level of the endogenous *Sox2* alleles (95% CI [6.1%-15.5%], based on RT-qPCR). Reporter integration does not affect endogenous *Sox2::mCherry* expression (Fig. S2A, reporter -116 kb) or the 3D genome organization of the locus (Fig. S2B, S5A-B).

We then induced SB hopping and monitored reporter fluorescence by flow cytometry. Compared to the non-hopped control, the resulting cell pool showed a broadened distribution of reporter fluorescence, indicating that the relocation at least in part affected reporter expression (Fig. 2B). We then split the full range of reporter expression values into six gates (P1-P6) and sorted pools of cells from each gate (Fig. 2B), and subsequently mapped the locations of the reporters in each pool. As a control, we also mapped the locations in unsorted cells. Since the integration patterns were reproducible between biological replicates (Fig. S8A), we combined the replicates for further downstream analysis.

As we previously observed with the arbitrary insert (Fig. 1C), reporter integrations in the control pool are mostly concentrated in a 2 Mb region surrounding the launch site (Fig. 2C, top track). In contrast, the cell pools with different expression levels yielded clearly distinct distributions (Fig. 2C-D, P1 – P6 tracks). Reporters with the highest activity (P1) were concentrated almost exclusively in the center of the SCR or close to the endogenous *Sox2* gene, while the second-most active reporters (P2) were more dispersed around these regions. Intermediate activity (P3 – P5) occurred predominantly near the launch site and between *Sox2* and SCR. Finally, silent (P6) reporters were almost exclusively located outside the *Sox2*-SCR TAD.

Because the expression of non-mobilized reporters overlaps the medium-level expression gates (P4 – P5, Fig. 2B), those gates contained many cells with an unhopped reporter and few new integrations. Sorting more cells did not fully solve this problem (Fig. S2C). To overcome this, we repeated the hopping experiments starting from a launch pad 51 kb upstream of *Sox2::mCherry* (-161 kb relative to the SCR) (Fig. 2E), where the reporter showed no measurable expression (Fig. S2A). Like the previous launch site, this reporter integration does not detectably affect endogenous *Sox2* expression (Fig. S2A), nor the 3D genome organization of the locus (Fig. S2B, S5C). As expected, this launch site makes it easier to detect hopping events in the medium-level expression bins, but harder in the silent (P6) bin (Fig. 2F-G). The integration pattern is reproducible between replicates (Fig. S8B) and closely resembles the pattern obtained from the -116 kb launch pad, but with more integrations recovered from the medium-level expression gates. A single hopping experiment from a second silent launch pad, as much as 715 kb downstream of the SCR, again confirms this pattern (Fig. S2D-E) and illustrates the advantage of silent launch pads, even when they are distant from the region of interest.

We converted these raw data into a reporter expression score track, which is the average expression level estimated in a sliding window across the locus (Fig. 2H, see Methods & Supplementary Text, section “Expression score”). This graph highlights that the SCR and the region immediately surrounding the endogenous *Sox2* gene are the most optimal locations for transcriptional activity. This is evident from the expression scores from the individual launch pads (Fig. S8E) as well as from the combined data (Fig. 2H). Precise fluorescence measurements in a panel of clonal cell lines with 36 unique integrations confirms this peak in activity at the center of the SCR and validates the pattern of expression scores inferred from the sorted pools. Reporter integrations in the center of the SCR display a 10-fold upregulation in reporter expression compared to the -116 kb launch pad position (Fig. S2G-H). Throughout the locus, reporter activity is independent of the orientation of the reporter (Fig. S2G, S2I). While the region between *Sox2* and SCR exhibits intermediate expression, there is a remarkable lack of reporter expression outside of the *Sox2*-SCR range, with a very sharp drop-off (within ~18 kb) on either side (Fig. 2H). Together, this detailed functional landscape points to a strong confinement of the realm over which the SCR is able to activate transcription. Note that the endogenous *Sox2* gene is still present in these experiments; below we will demonstrate that this gene strongly controls this landscape.

Within the *Sox2*-SCR TAD, the map reveals an intricate activity landscape with several local peaks of reporter expression (Fig. 2H). This landscape closely matches a map of SCR contact frequencies that was obtained in the absence of reporter integrations (18) (Fig. 2I top panel). Across the locus, reporter activity is correlated more strongly to the SCR contact frequency than the genomic distance to the SCR (Fig. 2J). Moreover, even for the fine-grained peaks and valleys between *Sox2* and the SCR (excluding these elements) the expression score from either the combined data (Fig. 2J) or the -161 kb launch pad (Fig. S2J-K) shows a good correlation with contact frequency to the SCR, suggesting that the variation in activity in this region is biologically meaningful. Maps of open chromatin (ATAC-seq (20)) and the histone mark H3K27ac (21), both thought to be features of regulatory elements (22), show only partial overlap with the fine pattern of reporter activity (Fig. 2I, bottom panel). These results indicate that reporter expression primarily follows contact frequency with the SCR, suggesting that these contacts are a major determinant of quantitative expression levels.

## Random deletion of the endogenous *Sox2* gene boosts reporter expression

**Fig. 3:**
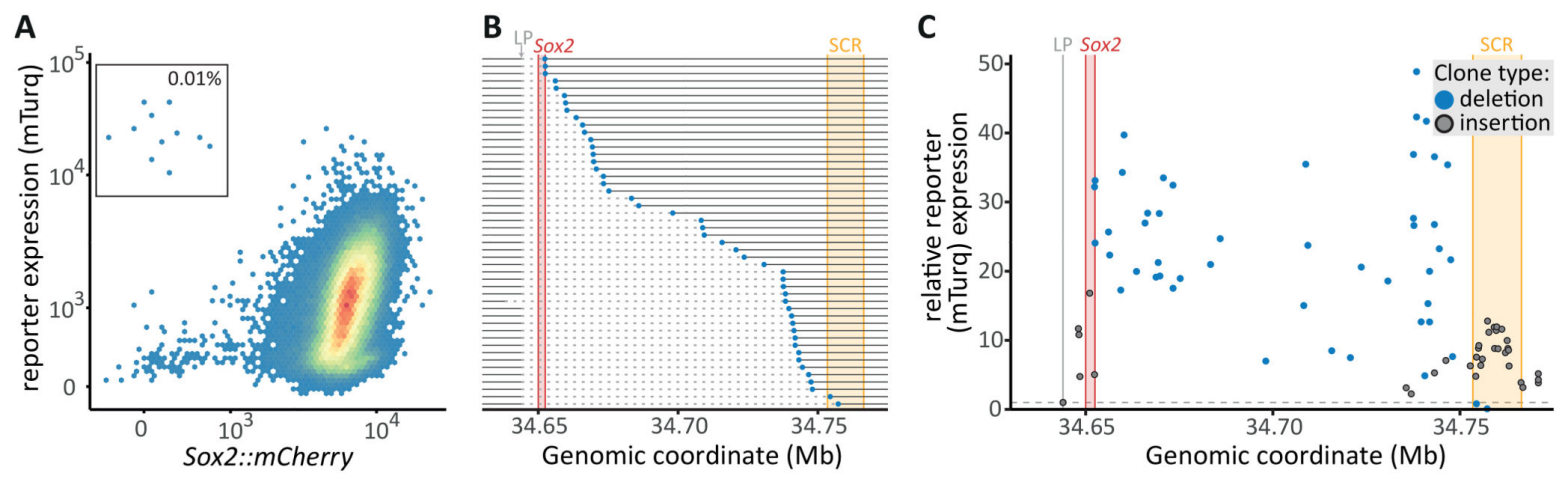
Hopping-induced deletions of the endogenous *Sox2* gene increase reporter expression. **(A)** Expression of *Sox2::mCherry* and Sox2P reporter (mTurq) after mobilization of SB from the -116 kb launch pad, first biological replicate (also shown in Fig. 2B). Box marks rare *Sox2::mCherry*-negative cells with high reporter expression. **(B)** Unique deletions inferred from mapping in *Sox2::mCherry*-negative clones (Fig. S3A), sorted by deletion size. Horizontal dashed lines indicate deletions. Dots indicate the locations of the re-insertions. Almost all deletions start at the launch pad (LP) position. **(C)** Relative reporter expression of clones with a hopping-induced deletion (blue dots, location corresponds to the end of the deletion) or regular insertion (grey, same data as in Fig. S2G, bottom). Clones from three biological replicates combined.

Close examination of the flow cytometry data of the transposase-transfected reporter cells (- 116 kb launch pad) revealed a small but distinct population of cells with high reporter expression but no *Sox2*::*mCherry* expression (Fig. 3A). We isolated a panel of clones with these unusual characteristics and mapped the SB location. In these clones, the left ITRs were still located at the launch site (upstream of *Sox2::mCherry*), while the right ITRs were linked to sequences distributed throughout the *Sox2* locus (Fig. 3B, S3A). This suggests that the genomic sequence between the launch site and integration site has been lost. Sanger sequencing confirmed the loss of the 129S1 allele in this region (Fig. S3B). We hypothesize that in rare instances, errors in the SB excision-reinsertion process can cause such a deletion, which can lead to the loss of the *Sox2::mCherry* gene.

Clones with such hopping-induced deletions exhibited high reporter expression, up to 49-fold higher than the -116 kb reporter clone (Fig. 3C). Reporters close to the SCR (within 20 kb) were expressed about 5-fold higher when a deletion was present, compared to regular insertions in the same area. Even the smallest deletions of 8.5 kb, removing only the endogenous *Sox2::mCherry* gene, resulted in substantially increased reporter expression. These data suggest that the endogenous *Sox2* gene strongly limits reporter expression when present. Other sequences within the *Sox2*-SCR range may further modulate this effect, indicated by the variable increases in expression across the deletions, which show no correlation with distance to the SCR (Spearman’s rho = 0.19, p = 0.19). In a few clones a fraction of cells lost reporter expression after sorting, causing a bimodal expression pattern (Fig. S3C). Clones sorted for loss of *Sox2*::*mCherry* expression with no reporter expression exhibited even larger deletions, extending beyond the SCR (Fig. S3D-F), confirming that the SCR is necessary for high reporter expression. In summary, these fortuitous, rare deletions suggested that the endogenous *Sox2* gene strongly reduces activity of the reporter gene throughout the *Sox2*-SCR range.

## *Sox2* gene strongly controls the regulatory landscape of the enhancer

**Fig. 4:**
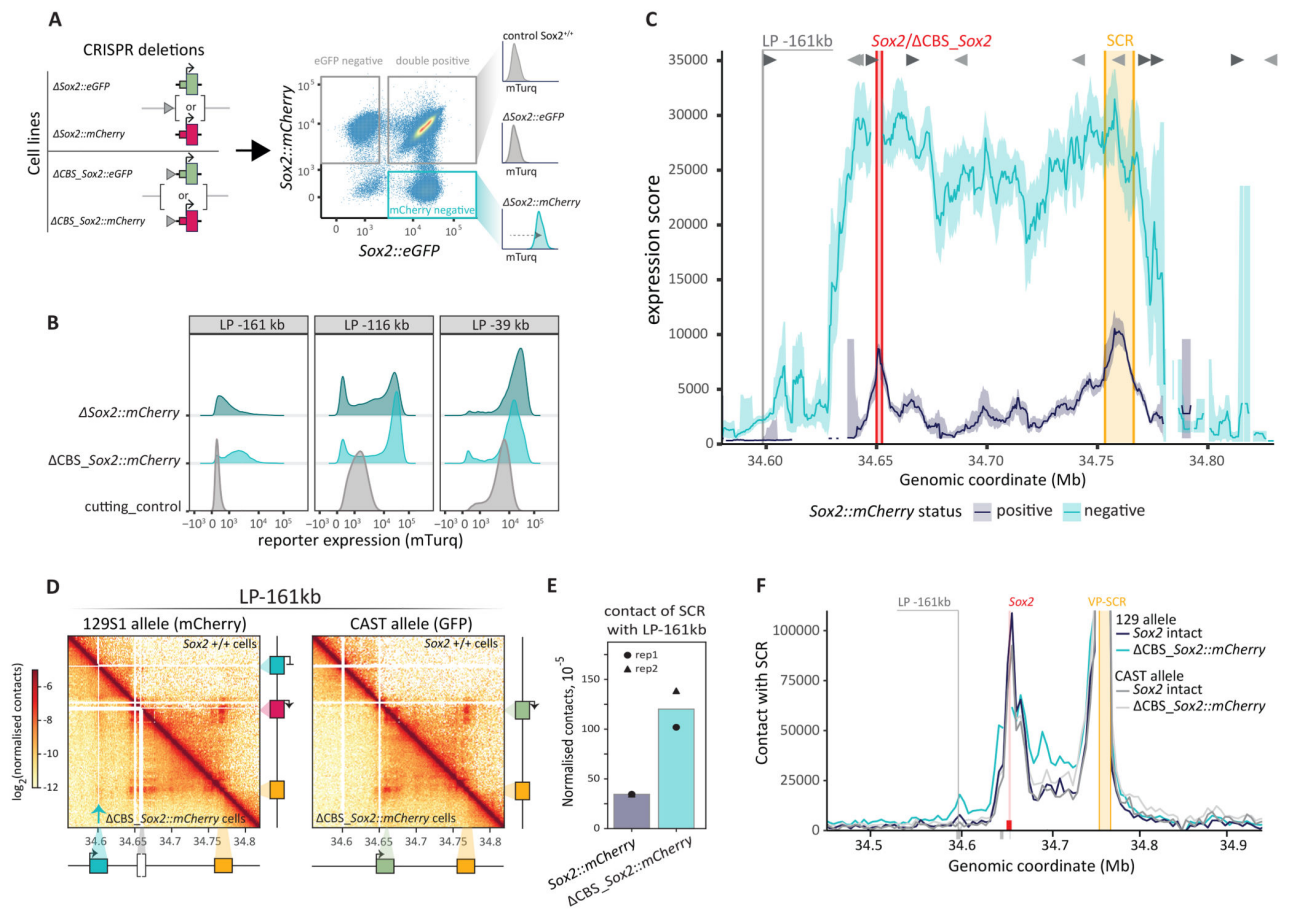
*Sox2* gene strongly confines the activation realm of the SCR. **(A)** Left panel: Cas9-mediated deletion of tagged *Sox2* alleles, with or without CBS (grey triangle); right panel: gating and analysis strategy. Negative fluorescence signal is due to background subtraction (see methods). **(B)** Distribution of mTurq reporter expression levels in three cell lines with reporter inserted in positions -161 kb, -116 kb, and -39 kb, after transfection with the indicated gRNAs, gated for eGFP+/mCherry-negative (Δ*Sox2::mCherry* and ΔCBS_*Sox2::mCherry*) or eGFP+/mCherry+ (cutting control) (see Fig. S4A for the other populations). **(C)** Expression score of reporter hopping from the -161 kb launch pad in a *Sox2::mCherry* intact cell line (same data as Fig. S2J, 2 biological replicates combined) or ΔCBS_*Sox2::mCherry* cell line (5 biological replicates combined). Calculated using a 5 kb window shifted in 500 bp steps and only plotted when the window contains 3 or more reporter integrations. Grey triangles indicate CBS positions and orientations. **(D)** Left panel: RCMC of the 129S1 allele (containing the reporter) with the -161 kb reporter integration, in the *Sox2::mCherry* intact (top right) or ΔCBS_*Sox2::mCherry* (bottom left) cell line. Data from two biological replicates combined, 1 kb resolution. Right panel: same as left panel for CAST allele. Genomic coordinates refer to the modified genome, same data as Fig. S7C, E. **(E)** Quantification of contact frequency between SCR and LP-161 kb in the presence or absence of *Sox2::mCherry*. **(F)** Virtual 4C contact profile (center of SCR as viewpoint), cell lines carrying reporter at -161 kb with *Sox2::mCherry* intact or ΔCBS_*Sox2::mCherry*, split by allele (all modifications are on the 129S1 allele). Data from two biological replicates combined, at 5 kb resolution, same data as Fig. S7C, E. Genomic coordinates according to mm10 genome, grey and red boxes mark insertions (landing pads and fluorescent tag of *Sox2*) and deletion of ΔCBS_*Sox2::mCherry*, respectively.

To verify this interpretation, we used CRISPR/Cas9 to delete the *Sox2* gene with (ΔCBS_*Sox2*) or without (Δ*Sox2*) the promoter CBS in three lines with mTurq reporters at - 161 kb (silent), -116 kb (low) or -39 kb (medium) relative to the SCR (Fig 4A). In each case, deletion of the *Sox2::mCherry* allele strongly upregulated the reporter in *cis*. Even the -161 kb reporter, normally inactive, became robustly expressed, indicating that *Sox2* can insulate upstream sequences from the SCR (Fig. 4B, S4A). We noticed that a proportion of cells lost reporter expression, which we explain in Supplementary Text, **section “Bimodality of reporter expression upon *Sox2* deletion”** (Fig. S4A-H). The highest reporter expression was not reached by the reporter closest to the SCR (-39 kb), but rather by the reporter closest to the native location of *Sox2* (-116 kb) (Fig. S4I). This suggests that the region around the endogenous *Sox2* gene is optimal for high expression, even when this gene including its promoter is deleted.

To further explore the promoter activation landscape of the *Sox2* locus in the absence of the competing endogenous *Sox2* gene, we conducted a reporter hopping experiment from the - 161 kb launch site in a ΔCBS_*Sox2::mCherry* clone, with an adapted gating strategy (see methods) (Fig. S4J-K). Across the *Sox2*-SCR interval the expression score is increased by 6-12-fold (interquartile range) when the endogenous *Sox2* gene is absent (Fig. 4C). While activity is now high across the locus, the highest levels are still in the regions around the SCR and around the deleted *Sox2* gene, indicating that these positions are optimal for high expression independent of the endogenous *Sox2* gene and its promoter. However, in absence of the gene, both optimal regions are broadened. In addition, high expression (more than half-maximum expression) spreads 17 kb further upstream and 6 kb further downstream when the *Sox2* is absent, and reporter expression is detectable up to 88 kb upstream of the deleted gene. We conclude that the *Sox2* gene strongly constrains both the level of activation of a competing promoter and the realm of influence of the SCR.

We wondered whether the change in the activation landscape reflects a change in the 3D genome organization of the locus. Region capture micro-C (RCMC) mapping (18) showed that the overall structure of the locus is preserved upon CBS_*Sox2::mCherry* deletion, but that there are several changes in contact (Fig. 4D). Firstly, the contact between the -161 kb reporter and the whole *Sox2*-SCR region is increased (Fig. 4D, **arrow**), suggesting that the reporter is now less insulated from the locus. Conversely, the SCR has increased contacts with the whole *Sox2*-SCR interval as well as the region upstream of the deleted *Sox2* gene (Fig. 4E, F). This spreading of SCR-contact appears to be dependent on the location of the reporter, since it extends less far when the reporter is in the -116 kb position (Fig. S5D-E), indicating that the reporter can affect the contact domain of the SCR in absence of the gene. The increase in SCR contact in the *Sox2*-SCR range, as well as the spreading upstream, might partially explain the increase in reporter expression in those locations. However, the increase in expression is substantially larger than the increase in contact frequency. This could be caused by further changes in the contact landscape upon each integration of the reporter, or it may or point to a non-linear relationship between contact and expression, as was noted before (9). Additionally, the increase in reporter expression score inside the SCR cannot be explained by changes in SCR contact (Fig. 4C, F), indicating that the removal of gene competition also affects the reporter in other ways than contact changes.

## Unbalanced competition is partially driven by the *Sox2* gene body

While the endogenous *Sox2* gene significantly restricts reporter expression, the reporter gene hardly affects the endogenous gene. First, the reporter inserted at -116 kb did not detectably affect *Sox2::mCherry* expression (Fig. S2A). Second, among all clonal lines with a hopped reporter, *Sox2::mCherry* expression is remarkably robust. Even in clones with a reporter in the center of the SCR (with ~10-fold higher expression than at -116 kb) *Sox2::mCherry* expression is reduced by only ~25% (Fig. S6A). This is extremely modest compared to the 3-30 fold upregulation of the reporter upon loss of *Sox2::mCherry* (Fig. 4C, Fig. S4I).

**Fig. 5:**
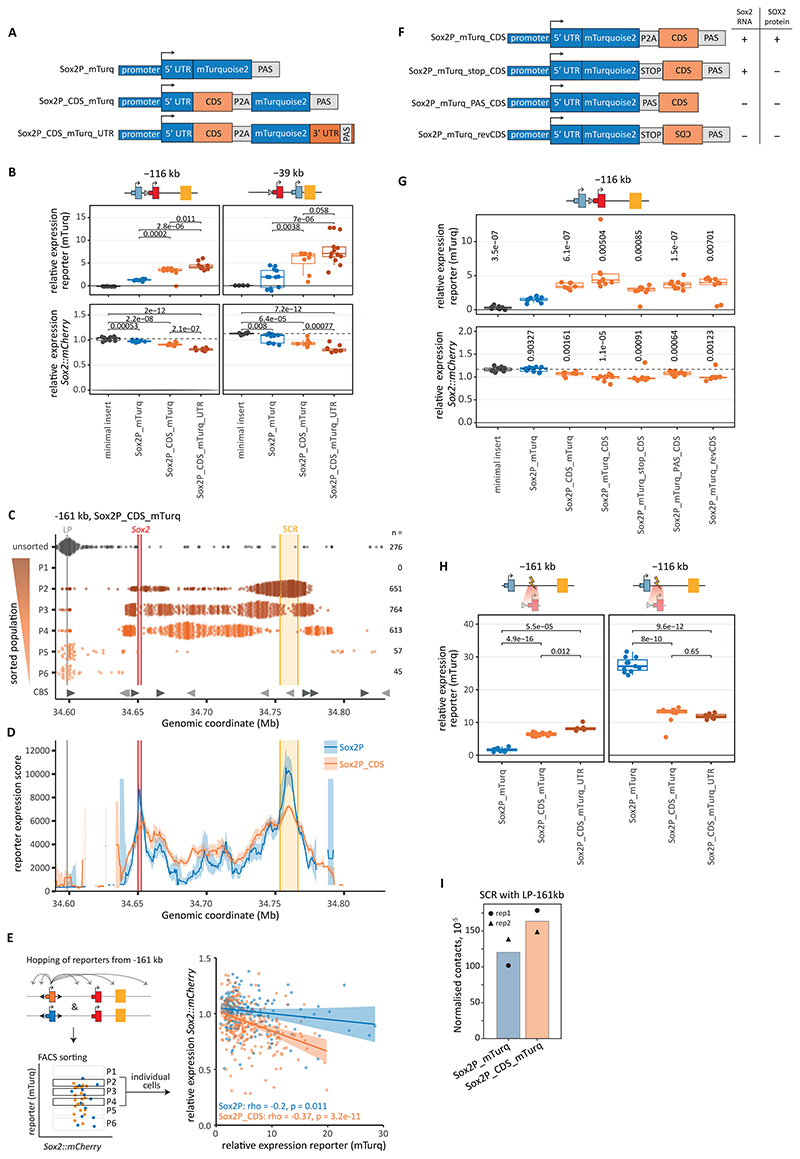
Unbalanced competition is partially driven by *Sox2* gene body. **(A)** Schematic of the original *Sox2* promoter reporter (Sox2P) and two coding sequence (CDS)-containing variants. PAS, polyadenylation signal; P2A, cleavage peptide. **(B)** Relative expression of the reporter (top) and endogenous *Sox2::mCherry* (bottom) for Sox2P, CDS-containing reporters, and a control construct lacking promoter and mTurq (“minimal insert”), integrated at the -116 kb and -39 kb launch pads in cells with intact *Sox2::mCherry*. Each dot represents a single clone. Reporter expression was background-subtracted, normalized to GFP, and then to the standard -116 kb Sox2P cell line measured on the same day. Dashed line on *Sox2::mCherry* indicates the median of the minimal insert. See Fig. S7A for raw expression values (not normalized to *eGFP*). Statistical significance calculated using Welch’s t-test. **(C)** Mapping of integration sites for sorted subpopulations (P1–P6; see Fig. S6F) and unsorted control cells. Visualization as in Fig. 2D; data from two biological replicates pooled. **(D)** Expression score of the Sox2P and Sox2P_CDS reporter across the *Sox2* locus (-161 kb launch pad); 5 kb window shifted in 500 bp steps. Expression score of the Sox2P reporter is same as in Fig. S2J. Shaded region indicates 95% confidence interval. Expression score is only plotted when the window contains 3 or more SB integrations. **(E)** Left panels: schematic of hopping (from launch pad -161 kb) and sorting Sox2P and Sox2P_CDS reporters based on reporter expression (P1-P6). Right panel: correlation between relative *Sox2::mCherry* and reporter (mTurq) expression from the Sox2P and Sox2P_CDS, from cells with reporter expression in the gates P2-P4 (see Fig. 2F and Fig. S6F). Relative expression was calculated as in (B), but per individual cell. Rho, Spearman’s correlation coefficient. Data are from two biological replicates combined. For visualization, the y-axis is cut at 1.5, excluding 8 outlier points (they are included in the correlation calculation); Fig. S6G shows complete data. (**F**) Design of modified CDS reporters with indication of the CDS being part of the reporter transcript (RNA) or translated (protein). STOP – translation stop codon; revCDS – reverse complement of the CDS. (**G**) Reporter and *Sox2::mCherry* expression for reorganized CDS constructs, as in (B). Statistical significance calculated using Welch’s t-test; for reporter expression every construct is compared to Sox2P, for *Sox2::mCherry* expression to ‘minimal insert’. See Fig. S7B for raw expression values (not normalized to eGFP). (**H**) As (B, top), but for at -161 kb and -116 kb in a ΔCBS_*Sox2::mCherry* cell line. See Fig. S7C for raw expression values (not normalized to eGFP). (**I**) Contact frequency, based on RCMC, between the SCR and -161 kb launch pad, containing the Sox2P or Sox2P_CDS reporter. Dots are independent biological replicates of the RCMC. Data for Sox2P_mTurq is same as in Fig. 4E.

We wondered what might give the endogenous *Sox2* gene this competitive edge. The reporter contained the exact promoter and 5’ UTR sequence from the *Sox2* gene, but it lacked the 1.0 kb coding sequence (CDS, *Sox2* has no introns) and the 1.1 kb 3’ UTR (containing the native polyadenylation signal [PAS]). Re-analysis of RCMC data (18) indicates that the SCR not only contacts the promoter of the endogenous *Sox2* gene, but also the gene body (Fig. S6B), indicating that the latter sequence might play a role. We therefore generated two new reporters in which we added the *Sox2* CDS alone or in combination with the 3’ UTR (Fig. 5A). We kept the *mTurq* sequence separated from the CDS by a P2A cleavage signal (23), so the stability of the fluorescent protein would not be affected by a fusion with the SOX2 protein.

We first tested these new reporters in the presence of *Sox2::mCherry*. Across multiple independent clonal cell lines, the CDS and CDS/3′UTR additions boosted reporter activity on average by 3-fold in the active -116 kb and -39 kb positions (Fig. 5B top, Fig. S7A). At position -161 kb, where the original reporter is silent (Fig. S2A), the CDS-containing reporter was also not expressed (Fig. S6C). Moreover, measurements of mRNA half-lives indicated that the CDS-containing transcripts are 1.5- to 4.7-fold less stable than the *mTurq*-only transcripts (Fig. S6D-E) indicating that differences in mTurq protein level underestimate the actual boosting effect of the CDS on the reporter transcription rate.

To assess if the CDS affects the ability of the reporter to be activated throughout the locus, we conducted hopping experiments with the CDS-carrying reporter. In the presence of the endogenous *Sox2::mCherry* gene, the sorted integrations of CDS-containing reporter follow a similar pattern as the original reporter (Fig. 5C). However, we did not detect any cells with the highest reporter expression (P1), causing flattening of the activity landscape near the SCR and the endogenous *Sox2* location (Fig. 5D, Fig S6F). Despite this, the CDS enhanced the reporter activity throughout the *Sox2*-SCR range (excluding *Sox2::mCherry* and SCR regions) (Fig. 5D). Note that, due to the reduced RNA stability, the mTurq levels give an underestimation of reporter transcription in presence of the CDS, and hence the expression levels are likely to be higher than measured.

We then asked whether active CDS-containing reporters also compete more strongly with the endogenous *Sox2* gene. Indeed, in the -116 kb and -39 kb positions, addition of the CDS to the reporter caused a 13-18% reduction in *Sox2::mCherry* expression, while inclusion of both CDS and 3’ UTR in the reporter caused a 21-31% reduction (Fig. 5B bottom, Fig. S7A). Next, in the pool of cells in which we mobilized the CDS-containing reporter from the -161 kb location, we compared the relative *Sox2::mCherry* and reporter expression of individual cells with active reporter (Fig. 5E). If the reporter competes with *Sox2::mCherry*, this should be reflected in reduced expression of the latter when the reporter is more active. With the reporter lacking the CDS, this effect is detectable but weak. However, when the CDS is present, reporter expression is higher which leads to more competition (Fig. 5E, S6G). Thus, across the random active locations sampled in this hopping experiment, the CDS boosts reporter expression and competition with the *Sox2* gene.

Next, we tested whether these effects of the CDS depend on its position within the reporter (by swapping the CDS and mTurq open reading frames), on its presence in the mature transcript (by inserting a PAS before the CDS), on its orientation (by replacing it with the reverse-complement sequence), or on its translation (by inserting a stop codon) (Fig. 5F, Fig. S6H). When tested in the lowly active -116 kb position, each of these modified CDS-reporters boosted reporter expression and dampened *Sox2::mCherry* expression (Fig. 5G, Fig. S6I, Fig. S7B). Although we cannot rule out that the PAS did not fully block transcription of the CDS, these results collectively indicate that the effect of the CDS is not dependent on production of mRNA in the sense orientation, nor on translation, nor on the exact position or orientation of the CDS. Since these characteristics are reminiscent of an enhancer, we examined whether the endogenous *Sox2* CDS has enhancer-like features. Indeed, in the native *Sox2* gene the CDS is specifically bound by the active chromatin mark H3K27ac and several transcription factors (Fig. S6J). Still, the CDS alone is not sufficient to stimulate the *Sox2* promoter (Fig. S6C), suggesting that it might function as a facilitator (24, 25) rather than as a classic enhancer.

To assess whether the CDS also boosts promoter activity when there is no competition with the endogenous *Sox2* gene, we first tested the CDS-containing reporters in positions -161 kb and -116 kb after deletion of *Sox2::mCherry*. In the -161 kb position, clonal cell lines with the CDS-containing reporters had 4-5 fold higher expression than those with the original reporter, indicating that the boost is independent of competition (Fig. 5H, Fig. S7C). With RCMC, we detected a modest increase in contact frequency between the SCR and the CDS-containing reporter compared to the original reporter (Fig. 5I, Fig. S6K), which might explain the facilitating effect of the CDS, although this needs to be explored further. In the - 116 kb position, the promoter-only reporters showed high but bimodal expression (Fig. 5H, Fig. S6L, Fig. S7C), as observed before (Fig. S4C, Fig. S4F-G). In contrast, the CDS-containing reporters exhibited stable but lower expression (Fig. 5H, Fig. S6L). This unimodal expression suggests that the CDS helps the promoter resist epigenetic silencing (Fig. S4H), making the reporter more similar to the endogenous *Sox2* gene. Repeating the experiment with the Sox2P_mTurq_stop_CDS reporter, which does not produce SOX2 protein, excluded the option that this lowered reporter expression is the result of a negative feedback loop on the SOX2 protein level (Fig. S6M, Fig. S7D). A possible explanation for the lower expression level is the lower RNA stability of the CDS-containing reporters. If there is a maximum to the activation or initiation rate of the *Sox2* promoter, all reporters in the -116 kb position setting might reach a similar transcription rate after *Sox2::mCherry* deletion. Then, the lower stability of the CDS-containing mRNA would result in a lower measured steady-state protein level. Such a saturation effect may also explain the dampened activity of the CDS-containing reporter when integrated in the SCR or near the endogenous *Sox2* gene (Fig. 5D). Despite this confounding effect, we conclude that the CDS can boost reporter expression in the absence of the endogenous *Sox2* gene.

## Discussion

Here we developed a powerful high-throughput “hopping” approach to create highly detailed quantitative maps of the activation potential of a native genomic locus, based on thousands of reporter integrations. We anticipate that this technology may also be used to systematically relocate other regulatory elements, such as enhancers, CBSs, synthetic sequences and other structural elements. It can also be used to generate series of genomic deletions from a few kb to more than 400 kb.

Earlier probing at low resolution in mouse embryos suggested that promoters respond to an enhancer whenever they are in the same TAD (11, 26). Systematic insertion of enhancers and promoters in heterologous genomic loci supported this notion, and pointed to a monotonous decay of promoter activity with increasing genomic distance to the enhancer (8, 9, 25). Our high-resolution functional maps of the native *Sox2* locus confirm that the influence realm of the SCR coincides with the TAD, but also reveal an intricate landscape in which pre-existing SCR contact frequency rather than genomic distance is a key determinant of promoter activity. Moreover, the region around the endogenous *Sox2* gene is a “sweet spot” for activation, even in the absence of the gene itself. Possibly, synergistic interactions between the SCR and enhancer-like elements surrounding the gene (16, 27) contribute to this, while the *Sox2* gene itself further confines this “sweet spot”.

Another finding is that deletion of the *Sox2* gene broadens the activation landscape and strongly increases expression of relocated promoters across the locus. This demonstrates that a gene can act as a gatekeeper, limiting the functional reach of its own enhancer. Various studies of native and artificial loci, both in *Drosophila* (28, 29) and mammals (30–32), showed competition between two promoters for activation by one enhancer. In each case, the enhancer preferentially activated the closest promoter. In contrast, our results show that the SCR strongly favors the native *Sox2* gene regardless of the reporter location. Part of this dominance is conferred by the ~1 kb CDS. Several previous reports suggested that enhancers can overlap coding exons (33–35), yet our data are more consistent with a role of the CDS as a facilitator of enhancer–promoter communication (24, 25), rather than as a conventional enhancer.

The broad variation in reporter expression observed in the hopping-induced deletion series suggests that additional elements throughout the locus are likely to contribute to the regulation of *Sox2* expression. This is in line with other studies (16, 27). Our reporter measurements after *Sox2::mCherry* deletion show that the CBS just upstream of *Sox2* is not sufficient to block reporter activation (Fig. 4B), but additional hopping experiments in combination with targeted deletions may uncover more subtle roles of this and other CBSs. More generally, our methodology to map the functional landscape of a large genomic locus provides new insights into the complex interplay between distant regulatory elements and should be widely applicable.

## Materials and Methods

### Cell culture

F121/9 (CAST/EiJ x S129/Sv) (RRID:CVCL_VC42) female mouse embryonic stem cell (mECS) F1 hybrid cell line (36) and derived clones were cultured in Serum+Lif+2i condition. Briefly, cells were cultured on gelatin-coated (0.1%) culture plates in Glasgow minimum essential medium (Sigma-Aldrich, G5154) supplemented with 15% fetal bovine serum (Thermo Fisher Scientific, 10270-106), 1% L-glutamine (Thermo Fisher Scientific, 25030024), 1% sodium pyruvate (Thermo Fisher Scientific, 11360-70), 1% MEM non-essential amino acids (Thermo Fisher Scientific, 11140-50), 1% penicillin-streptomycin (Thermo Fisher Scientific, 15070063), 100 μM ß-mercaptoethanol, 10 x 4 U leukemia inhibitory factor (LIF; esg1107, Millipore), 1 μM MEK inhibitor PD0325901 (Mirdametinib, MedChemExpress), 3 μM GSK-3β inhibitor CHIR99021 (Laduviglusib, MedChemExpress) in 5% CO_2_ at 37 °C. Cells were passaged every 2 days.

Mycoplasma contamination was ruled out by regular testing (#LT07-318; Lonza).

### Genetic engineering of the *Sox2* locus: Tagging endogenous *Sox2* alleles

The alleles of the *Sox2* gene were tagged by direct fusion with eGFP and mCherry using CRISPR/Cas9 mediated homologous recombination. We used a previously described plasmid for targeting *Sox2* (*Sox2* sgRNA, Addgene #175553) together with donor plasmids designed to include eGFP or mCherry in between two homology arms (200 bp homologies) for the *Sox2* gene. In brief, 1x10^6^ F121/9 mESCs were transfected with 1 μg of *Sox2* sgRNA and 2 μg of repair template (1 μg of eGFP + 1 μg of mCherry) using 9 μL Lipofectamine2000. Cells were plated in Serum+Lif+2i medium together with a DNAPKcs inhibitor (M3814, MCE, HY-101570) and cultured for two days. Subsequently, eGFP+/mCherry+ cells were sorted sparsely into a 24-well plate (cultured without inhibitor) and resulting colonies were picked and genotyped by PCR. Then they were re-measured by flow cytometry (LSRFortessaTM) using the following lasers and filters: eGFP 488nm BL[B] 530/30, mCherry 561nm YG[D] 610/20. Allele-specific genotyping of *Sox2* was performed by PCR amplification from the fluorophore to the genome across CAST/129S1 SNPs (primers: EMp324, EMp325, MEP68). A clone with the CAST allele *Sox2* gene fused with eGFP and the 129S1 allele *Sox2* gene fused with mCherry was selected as the parental clone.

### Genetic engineering of the *Sox2* locus: Establishing launch pads

We cloned the vector pME015 containing an mouse *PGK* promoter driving the expression of a double selection marker HyTK (HyTK amplified from plasmid #11684, Addgene), flanked by FRT and F3 heterotypic recombination sites embedded between Sleeping Beauty 5’ and 3’ inverted terminal repeats (ITRs). For establishing the -116 kb launch pad, we used the Alt-R™ CRISPR-Cas9 system from IDT and designed a crRNA that directs cutting 6 kb upstream (-116 kb from SCR) of the endogenous *Sox2*. The crRNA was assembled into an RNP together with the AltR CRISPR tracrRNA (1073189, IDT) and Cas9 nuclease (1081058, IDT). We transfected 50x10^3^ parental cells (*Sox2* eGFP+/mCherry+) with the RNP and the PCR product using lipofectamine CRISPRMAX Cas9 Transfection Reagent (CMAX00001, Thermo Fisher Scientific) according to the manufacturer’s protocol. Two days after transfection, Hygromycin was added to the medium (200 μg/mL, 10687010, Invitrogen) to select for HyTK-expressing cells. We picked colonies 6 days later and screened clones for cassette integration by PCR and Sanger sequencing.

We created a launch pad in-between *Sox2* and SCR (-39 kb relative to SCR) by CRISPR/Cas9 mediated homology directed repair. For this, we added 1 kb homology arms of the target region upstream and downstream of the HyTK construct (pME015) to generate plasmid pME024, assembled the crRNA with the tracrRNA and Cas9 protein as explained above, and co-transfected the RNP complex together with the pME024 repair template into parental cells (*Sox2* eGFP+/mCherry+). Similar as above, cells were selected with Hygromycin and colonies were picked and genotyped by PCR and Sanger sequencing.

### RMCE donor plasmids: minimal insert

We generated 2000 random 50 nucleotide sequences in silico with a GC content of 50% and scanned them by FIMO for transcription factor binding sites (37). We selected one sequence without any transcription factor binding sites, added FRT and F3 sites to the start and end of the sequence and ordered it as an DNA ultramer from IDT (sequence in Table S5). We amplified this sequence by PCR with primers that have 40 bp overlap to the digested ends (BamH1+XhoI) of the target plasmid (pCR Zero, #120275, Addgene) (primers: MEP190, MEP191). Finally we used Gibson assembly to assemble the linearized pCR-Zero vector together with the amplified PCR product according to the manufacturer’s protocol. Two μL of Gibson assembly mix were transformed into 50 μL DH5α (NEB, C2987H) chemically competent cells. Clones were screened by colony PCR and validated by Sanger sequencing.

### RMCE donor plasmids: *Sox2* reporter construct

We amplified 2346 bp of the *Sox2* promoter (containing the 5’UTR) from a fosmid (Wl1-1017O07, position mm9: chr3:34535173-34570689, kindly provided by Luca Giorgetti’s lab) by PCR using Phusion Polymerase (F-530L, Thermo Fisher Scientific) together with DMSO and GC rich buffer (primers: MEP145, MEP150). The resulting PCR product was cloned into pCR Zero vector (#120275, Addgene) digested with StuI (R0187S, NEB) and transformed into 50 μL DH5α (C2987H, NEB) chemically competent cells. Resulting colonies were picked and genotyped by PCR and validated by Sanger sequencing. To generate the Sox2P-mTurquoise2 plasmid, the *Sox2*-promoter-containing pCR Zero plasmid was digested with Xbal + SalI-HF (R3138S, NEB) and ligated (T4 Ligase 5 U/μL, 9015-85-4, Roche) with the Xbal+SalI-HF-digested mTurquoise2-plasmid (#118617, Addgene), resulting in a plasmid containing the *Sox2* promoter with the mTurquoise2 fluorophore. Finally, an SV40 polyA signal was cloned 3’ of the mTurquoise2 (primers: MEP171, MEP172) and F3 and FRT heterotypic Flp-recombinase sites were added by PCR (primers: MEP147, MEP177), resulting into the final pME034 plasmid. The plasmid was validated by Sanger sequencing. Primer sequences can be found in Table S2.

### RMCE donor plasmids: *Sox2-*CDS reporter construct

We first generated two additional *Sox2* reporters containing the *Sox2* coding sequence (957 bp) and either the endogenous *Sox2* 3’UTR or the same polyA signal (SV40) as the original Sox2P reporter construct (pME034). We linearized the reporter plasmid (pME034) with SalI-HF & EcoRV-HF, and PCR amplified the mTurquoise2 gene ± polyA signal from pME034 (primers: MEP224, MEP225, MEP231) and *Sox2*-3’UTR (primers: MEP226, MEP230) and *Sox2*-CDS (primers: MEP222, MEP223) from the same fosmid as before (Wl1-1017O07). A P2A sequence with linker was added by the forward primer for the mTurquoise2 gene (MEP224). We used Gibson assembly to create either the *Sox2*-CDS-polyA(SV40) or the *Sox2*-CDS-3’UTR plasmids. Next, we checked the resulting plasmids by colony PCR and subsequent Sanger sequencing. The final plasmids were named pME040 (FRT_Sox2P_CDS_mTurq_3’UTR_F3) and pME041 (FRT_Sox2P_CDS_mTurq_polyA_F3).

To generate the second set of seven CDS-containing reporters, we first linearized pME034 as before by digestion with SalI-HF & EcoRV-HF and PCR amplified from pME034 the mTurquoise2 gene (primers: CMP122, CMP123 or CMP133) and polyA signal (primers: CMP124, MEP231) and from pME041 the *Sox2-*3’UTR (primers: CMP134, 135). An SbfI restriction site was added on the mTurquoise2 reverse primers and the polyA/3’UTR forward primers. We used Gibson assembly to create the intermediate plasmids pCM037 (FRT_Sox2P_5′UTR_mTurq_SbfI_PAS_F3) and pCM041 (FRT_Sox2P_5′UTR_mTurq_SbfI_3’UTR_F3). Next, we generated the following CDS-containing inserts by PCR amplification from pME040, with extra sequences on the forward primers: P2A-CDS (primers: CMP125, CMP126 or CMP136), STOP-CDS (primers: CMP127, CMP126 or CMP136), STOP-revCDS (primers: CMP131, CMP132). The reverse primers contain overlaps to the polyA signal (CMP126, CMP132) or 3’UTR (CMP136). We linearized pCM037 and pCM041 by digestion with SbfI-HF and used Gibson assembly to create the following plasmids:

- pCM038 (FRT_Sox2P_5′UTR_mTurq_P2A_CDS_PAS_F3)
- pCM039 (FRT_Sox2P_5′UTR_mTurq_STOP_CDS_PAS_F3)
- pCM041 (FRT_Sox2P_5′UTR_mTurq_STOP_revCDS_PAS_F3)
- pCM043 (FRT_Sox2P_5′UTR_mTurq_P2A_CDS_3’UTR_F3)
- pCM044 (FRT_Sox2P_5′UTR_mTurq_STOP_CDS_3’UTR_F3)

pCM045 (FRT_Sox2P_5′UTR_mTurq_PAS_CDS_3’UTR_F3) was generated by assembling polyA signal (from pME034, primers: CMP137, CMP128) and CDS (from pME041, primers: CMP129, CMP136) into the linearized pCM042. pCM040 (FRT_Sox2P_5′UTR_mTurq_PAS_CDS_F3) was generated directly by assembling mTurquoise2+polyA (from pME034, primers: CMP122, CMP128) and CDS (from pME041, primers: CMP129, CMP130) with the F3 sequence on the reverse primer, into the SalI-HF & EcoRV-HF linearized pME034. Resulting plasmids were verified by colony PCR, Sanger sequencing and nanopore whole plasmid sequencing. Primer sequences can be found in Table S2.

### Recombination-mediated cassette exchange (RMCE)

Inserts were loaded into the SB cassette by recombination-mediated cassette exchange (RMCE). 300,000 cells were transfected with 0.5 μg of Flp recombinase-encoding plasmid (Addgene #13787) and 2 μg of donor plasmid, using 7.5 μL lipofectamine 2000 (Invitrogen, 11668019). Two days after transfection, pools were seeded sparsely and ganciclovir (2.5 μg/mL) was added to the medium to kill HyTK-expressing cells. Resistant colonies were picked, expanded, and screened by PCR. To replace the SB insert in a Sox2P-containing clone derived by SB hopping or CRISPR editing, we first exchanged the Sox2P reporter for the original HyTK cassette by co-transfecting 400,000 to 500,000 cells with 0.5 μg of Flp recombinase-encoding plasmid and 2 μg of a HyTK-containing plasmid (pME015). Two days after transfection, pools of HyTK-expressing cells were selected by adding Hygromycin to the medium. For -161 kb ΔCBS_*Sox2::mCherry* and -116kb ΔCBS_*Sox2* HyTK-containing clones were derived by FACS sorting mTurq-/mCherry-/eGFP+ cells from the HyTK-containing pools. Next, various constructs were loaded into these HyTK-containing pools (Fig. 5B, Fig. 5H, Fig. S6C) or clones (Fig. 5G, Fig. S6I, Fig. S6M) using RMCE, as explained above.

Cells containing the minimal insert, Sox2P-reporter, or the CDS-containing reporter constructs in different launch pads (-39 kb, -116 kb, -161 kb) were generated by RMCE and selected with ganciclovir. Clones were obtained by colony picking or single cell sorting into 96 well plates. Subsequently, clones were genotyped by PCR. Only clones containing the right insert and genomic background were measured by flow cytometry (*Sox2::mCherry, Sox2::eGFP*, reporters) and used for downstream analysis.

### Establishing proof-of-principle cell line

For a different project we used a plasmid containing a stretch of 200 random nucleotides (free of any CTCF motifs), followed by a CTCF binding site flanked by LoxP sites (kindly provided by Luca Braccioli, Elzo de Wit group). We PCR amplified the insert with flanking F3/FRT sites (primers: MEP74, MEP75) and cloned the resulting PCR product into StuI blunt cut pCR-Zero vector, creating donor plasmid pME013. Next, we used RMCE to integrate the insert into the -116 kb launch pad of *Sox2* eGFP+/mCherry+ F121/9 mESCs, resulting in a cell line called CBS16. To generate a ΔCTCF control cell line (CRE6), we transfected the cells with a CRE-Puro plasmid (pJK14-946-CRE-Puro), selected with Puromycin and seeded surviving cells as single cells in 96 well plates by FACS (BD FACSAriaTM Fusion Flow Cytometer). Resulting clones had an insert of 282 bp in between FRT/F3 sites and were genotyped for the floxed CTCF motif by PCR and validated by Sanger sequencing. Those cells were used for the proof of principle experiment. The sequence of the resulting insert (200 arbitrary nucleotides and one loxP site) can be found in Table S5. Primers can be found in Table S2.

### SB hopping

For each replicate and cell line, SB hopping was induced by transfecting 2.5 to 5 million cells with the bicistronic plasmid (pME07) encoding SB transposase SB100x and the human nerve growth factor receptor (LNGFR), using 15 μL Lipofectamine 2000 (Invitrogen, 11668019) and 5 μg of plasmid per million cells. As a control, 0.5 million cells were transfected with a similar bicistronic plasmid encoding PiggyBac (PB) transposase and LNGFR (pLD042). After 30 hours, transfected cells were enriched using MACS MS columns (130-042-201, Miltenyi Biotec) and LNGFR MicroBeads (130-091-330, Miltenyi Biotec) according to the manufacturer’s protocol.

One week after transfection, 0.2 million SB-transposase-transfected cells were set apart as an unsorted pool and the remaining cells were sorted by FACS (BD FACSAriaTM Fusion Flow Cytometer) into pools for populations based on mTurq expression level, using the following lasers and filters: mTurq: 442nm V[F] 470/20, eGFP: 488nm BL[B] 530/30, mCherry: 561nm YG[D] 610/20 (see Fig. 2B for the exact gates). PB transposase transfected cells served as mock-treated FACS control. In the first biological replicate of the -116 kb Sox2P-reporter (Fig. 2B, Fig. 3A) cells were only selected based on mTurq level, in all later replicates we selected only *Sox2::mCherry*-positive cells for all *Sox2::mCherry* containing cell lines and only *Sox2::mCherry*-negative cells for the ΔCBS_*Sox2::mCherry* cell line. For the -161kb_Sox2P ΔCBS_*Sox2::mCherry* cell line, the first two replicates were sorted into the same expression gates the *Sox2::mCherry* containing cell lines (i.e. P1-P6). However, to better distinguish between the different high expression levels, we split P1 and P2 up into a High and Low sub-gate (P1 H, P1 L, P2 H, P2 L) for replicates 3 (P1) and 4 (P1 and P2) (see Fig. S4J for the exact gates). Cells were sorted into up to 5 pools of up to 2000 cells per pool. To increase the number of integrations, we also sorted one pool of 50,000 cells per replicate for one replicate of -116kb_Sox2P population P3-P5 and for two replicates of -161kb_Sox2P_ΔCBS_*Sox*2 P2-P6 (P6 rep2 only 5200 cells). Sorted pools were expanded and for the small pools (up to 2000 cells) crude lysates were obtained from a full 96 well plate by lysing with 50 μL DirectPCR Lysis Reagent (102-T, Viagen Biotec) supplemented with 100 μg/mL proteinase K, and incubating 2.5 h at 55 °C and 45 min at 85 °C. For the 50,000 cell pools and the unsorted cell pools, gDNA was extracted after expansion using the ISOLATE II Genomic DNA Kit (BIO-52067, Bioline).

### SB hopping: establishing clones

To generate a panel of clonal cell lines with active reporters, we FACS sorted single cells either from previously sorted P1 and P2 pools (biological replicate 1) or directly from the full pool of SB-transposase-transfected cells (replicates 2 and 3, only deletion clones). mCherry-positive and mCherry-negative cells were sorted to obtain clones with hopped insertions and hopping-induced deletions, respectively. When sorting from the full pool of SB-transfected cells we also gated for high (P1) mTurq expression. The cell lines with a reporter integration at -161 kb (chr3: 34598479) and +715 kb (chr3: 3547841) were isolated in an earlier SB hopping experiment (not described here), by sorting out mTurq-negative cells from a pool of SB-transposase-transfected cells (from the -116 kb reporter cell line). Sorted clones were expanded and crude lysates were obtained from a full 96 well plate, as for the cell pools. Deletion clones presumably derived from one hopping event (from the same experiment with identical location and orientation) were averaged.

### Hopping-induced deletion clones

Hopping induced deletion clones were sorted based on the absence of *Sox2::mCherry* and the reporter being expressed or not (Fig. S3D). Sorted clones were expanded and DNA was harvested by direct lysis (see above). Deletions were mapped via Tn5-tagmentation and some clones with low read counts were further verified by targeted PCR amplification. We used PCR (primers: CMP116-121) to amplify three regions containing annotated SNPs on the CAST allele and Sanger sequencing to evaluate which allelic fragment(s) got amplified. By examining the Sanger sequencing traces, we could categorize if and which allele got deleted (Fig. S3B).

### Tagmentation mapping

SB integrations were mapped by amplifying and sequencing the ITR-genome junction using a tagmentation-based approach, based on (19). The procedure was similar to (38), but using the following SB-ITR specific primers: MEP009 (5′ ITR, reverse) or LD027 (3′ ITR, forward) for the linear enrichment and MEP011 (5′ ITR, reverse) or MEP034 (3′ ITR, forward) for PCR1. Input for one tagmentation reaction was either 100ng gDNA or 3 μL crude lysate. The 72 °C extension times of all PCRs were increased to 1 min. In addition, the composition of the linear enrichment PCR was changed to 10 μL of tagmented DNA, 2 μL of 1 μM primer, 4 μL dNTPs (10mM), 8 μL 5x Phusion® HF Buffer (NEB), 0.5 μL Phusion® HS Flex polymerase (2 U/μL - NEB), in a final volume of 40 μL. For all clones and some experiments with sorted pools (proof-of-principle experiment, third replicate of -116kb_Sox2P) we halved this reaction to a final volume of 20 μL. For the unsorted control pools, 10 separate tagmentation reactions and library preparations were performed, usually using one set of sequencing indices. For each of the large sorted pools (with 50,000 cell complexity), 20 separate tagmentation reactions and library preparations were performed with one set of sequencing indices, to obtain more exhaustive mapping, comparable to the small sorted pools. Libraries were sequenced with 150 bp paired-end reads using either an Illumina MiSeq system (reagent kit v2) or Illumina NextSeq 550 system (mid-output kit) including 10% of PhiX spike-in.

### CRISPR deletion endogenous *Sox2* gene

To delete the endogenous *Sox2* gene, we designed two gRNAs targeting upstream of the CTCF site, one at the start of the *Sox2* promoter (not targeting the reporter) and three downstream of the *Sox2* gene (one in the 3’ UTR, two after). gRNAs were cloned into an expression plasmid containing mCherry. One guide per region was selected (up1c, up2c, do1c) and 0.5 million cells were transfected with 1 μg Cas9 plasmid (pX458, Addgene #48138) and 1.5 μg gRNA plasmid (equimolar pool of multiple guides, or single control plasmid) using 7.5 μL lipofectamine 2000. Single gRNA plasmid controls and a cutting control (gRNA targeting a non-relevant gene:*Tdgf1*) were used to assess gRNA specific phenotypes that are not related to the deletion of the endogenous *Sox2* gene. One day post transfection, cells were sorted for the presence of the plasmids (mCherry & eGFP). Seven days after transfection, *Sox2* and reporter expression were measured in these cells by flow cytometry (BD FACSAria™ Fusion).

*Sox2::mCherry*-deleted pools and clones were generated in an earlier experiment, with the following differences: we transfected pools with multiple gRNAs per location, used the Cas9 plasmid pX330, and sorted pools of *Sox2::mCherry* negative cells with high reporter expression as pools more than three weeks after transfection. *Sox2::mCherry* negative, reporter-high clones were either isolated at the same time (-116 kb ΔCBS_*Sox2*), or isolated later from the sorted pools by single cell dilution (-116 kb *ΔSox2*) or by FACS sorting *Sox2::mCherry*-negative mTurq-positive cells (-161 kb ΔCBS_*Sox2*, -39 kb ΔCBS_*Sox2*). We isolated gDNA or crude lysates (as before) for all clones and performed a PCR across the intended deletion (*ΔSox2*: CMP075+CMP081; ΔCBS_*Sox2*: CMP077+CMP081). In addition, we performed a PCR from the intact GFP or 3’ UTR to the downstream region (CMP024+CMP081 or CMP080+CMP081) to confirm that the *Sox2::eGFP* allele was not inverted. Clones where either PCR failed were excluded from all analyses. All gRNA and primer sequences can be found in Table S1 and S2.

### CRISPR deletion SCR

We cloned two previously published gRNA sequences (13) targeting 5′ or 3’ of the SCR into a Cas9 expressing px330 backbone (#158973, Addgene) according to the protocol published by the Zhang lab (39). Used gRNA sequences were reported to work in mESCs in an earlier publication (13). Resulting plasmids were verified by Sanger sequencing and transfected into *Sox2* eGFP+/mCherry+ F121/9 mESCs. In brief, 0.5 million cells were transfected with 0.5 μg 5’SCR-sgRNA plasmid and 0.5 μg 3’SCR-sgRNA plasmid using Lipofectamine2000. Cells were analyzed for eGFP and mCherry expression by FACS (LSRFortessaTM) after 5 days of culturing using the following lasers and filters: eGFP BL[B] 530/30, mCherry YG[D] 610/20. gRNA sequences used for SCR deletion can be found in Table S1.

### Flow cytometry

Allele-specific *Sox2* expression and reporter fluorescence of clones and pools were measured on a BD LSRFortessa™ equipped with a High Throughput Sampler (HTS) for 96-well plate acquisition. Fluorescence was detected using the following laser/filter settings: eGFP (488 nm, 530/30), mCherry (561 nm, 610/20), and mTurquoise2 (405 nm, 470/20). Single-color controls confirmed no spillover of mTurquoise2 into the GFP channel. Single cells were gated in FlowJo, and fluorescence data were exported as .fcs files for analysis in R using the flowCore package (40).

Cell sorting was performed on a BD FACSAria™ Fusion using the same filter configuration, but mTurquoise2 was excited with a 445 nm laser for improved performance. To correct for spectral overlap, 1.6% of the 470/20 signal (mTurquoise2) was subtracted from the 530/30 GFP channel. Automatic background signal subtraction resulting from the fluid between cells is applied by the FACS machine.

### CTCF ChIP

F121/9 (CAST/EiJ x S129/Sv) *Sox2* /eGFP+/mCherry+ were cultured in Serum+Lif+2i condition. For chromatin preparation, 10 million cells (per condition) were cross-linked with a final concentration of 1% formaldehyde for 10 min. The cross-linking reaction was quenched with 2.0 M glycine (0.2 M final concentration). The cross-linked cells were then lysed and sonicated to obtain approximately 300 bp chromatin fragments with a Bioruptor Plus sonication device (Diagenode). For ChIP assays, antibodies were first coupled with Protein G dynabeads (10003D, Thermo Fisher Scientific), and the sonicated chromatin was then incubated overnight at 4 °C. After incubation, the beads were washed, the captured chromatin was eluted and cross-linking was reversed. The released DNA fragments were purified with the MinElute PCR Purification kit (28004, Qiagen). The ChIP experiment was performed with the following antibody: CTCF (Millipore Cat# 07-729, RRID:AB_441965; 5 μL per ChIP). The purified DNA fragments were prepared according to the protocol of the KAPA LTP Library Preparation kit (KR0453, Roche) before sequencing. All ChIP-seq libraries were sequenced with single-end 150 bp cycle mode on an Illumina NextSeq 550 (mid-output kit).

### RNA stability measurements

Reporter cell lines (Sox2P_mTurq, Sox2P_CDS_mTurq, Sox2P_CDS_mTurq_UTR), each with integrations at -116 kb relative to the SCR, were treated with 10 μg/ml (final concentration) ActinomycinD (Sigma Aldrich, SBR00013). Cells were harvested in 1 ml TRIzol™ (Invitrogen, 15596018) at multiple timepoints post-treatment: 0, 0.5, 1, 2, 4, 6, 8, 10, and 12 hours. RNA was extracted, DNase-treated, and reverse transcribed using Tetro Reverse Transcriptase with random hexamers (GC Biotech, BIO-65050). cDNA was diluted 1:10 and used as input for qPCR. qPCR was performed with SensiFAST SYBR Green No-ROX (GC Biotech, BIO-98020), targeting *GFP* (CAST–Sox2), *mCherry* (129–Sox2), *mTurquoise2* (reporter), total *Sox2, GAPDH*, and *β-Actin. GFP* and *mTurq* primers were designed to specifically amplify the intended target and checked by PCR. Reactions (10 μl) included 2.5 μl diluted cDNA, 2.5 μl primer mix (800 nM each), and 5 μl SYBR mix. Cycling conditions: 95 °C for 5 s, 60–65 °C for 10 s, 72 °C for 10 s, for 45 cycles. For Sox2P_mTurq and Sox2P_CDS_mTurq, we performed two biological replicates for timepoints 0, 0.5, 1, 2, 4, and 6 hours, and two additional replicates for timepoints 0, 1, 2, 4, 6, 8, 10, and 12 hours. For Sox2P_CDS_mTurq_UTR, two biological replicates were performed at 0, 1, 2, 4, 6, 8, 10, and 12 hours. All qPCR reactions were run in technical triplicates. Primer sequences can be found in Table S4.

### Region capture Micro-C

Region capture Micro-C (RCMC) was performed as previously described (18). In brief, the protocol consisted of crosslinking, MNase digestion, end labeling and proximity ligation, reverse crosslinking and dinucleosome purification, library prep and amplification, and target enrichment.

For each condition, approximately 25M cells were trypsinized, pelleted, and resuspended in PBS at a final concentration of 1M cells/mL. Disuccinimidyl glutarate (Thermo Fisher #20593) was added to a final concentration of 3mM and cells were fixed with gentle rotation for 35 minutes. 16% paraformaldehyde (Thermo Fisher #28908) was added to the suspended cells and fixation was continued for 10 minutes. The crosslinking reaction was quenched by adding 2M Tris pH 7.5 (K-D Medical #RGE-3370) to a final concentration of 0.375M. Cells were pelleted, washed with PBS, and aliquoted to pellets of 5M cells.

Crosslinked cell pellets were resuspended in Micro-C buffer #1 (50mM NaCl, 10mM Tris, 5mM MgCl2, 1mM CaCl2, 0.2% NP-40 alternative (Millipore #492018), 1x protease inhibitor cocktail (Sigma-Aldrich #5056489001)) and incubated on ice for 20 minutes to extract nuclei. Nuclei were then treated with 50 units of micrococcal nuclease (MNase, Worthington Biochem #LS004798) per 5 million cells and incubated at 37 °C for 20 minutes to digest chromatin into primarily nucleosome-sized fragments. The optimal concentration of MNase was determined by a titration experiment on additional cells crosslinked as above. MNase digestion was quenched by adding 500mM EGTA to a final concentration of 4mM and incubating at 65 °C for 10 minutes.

Digested pellets were washed twice with Micro-C buffer 2 (50mM NaCl, 10mM Tris-HCl pH 7.5, 10mM MgCl2, 100 μg/mL bovine serum albumin (Sigma Aldrich #B8667)), then resuspended in end repair buffer (as Micro-C buffer 2, plus 2mM ATP and 5mM dithiotreitol) and treated with T4 polynucleotide kinase (New England Biolabs M0201) for 15 minutes at 37 °C. 50U of Klenow fragment (NEB #M0210) was added to the suspended nuclei, and tubes were incubated for 15 more minutes at 37 °C. The mixture was then supplemented with biotin-dATP and biotin-dCTP (Jena Bioscience #NU-835-BIO14 and #NU-809-BIOX), dGTP and dTTP (Jena Bioscience #NU1003 and #NU1004) to a final concentration of 66μM each, 10X New England Biolabs T4 Ligase buffer (#B0202S) to final concentration of 0.34X, and additional BSA to maintain a concentration of 100μg/mL, and incubated at 25 °C for 45 minutes. EDTA at a final concentration of 30mM was used to quench the reaction, and the mixture was incubated at 65 °C for 20 minutes to fully inactivate the enzymes. This step produced chromatin fragments with blunt ends labeled with biotinylated nucleotides.

Labeled nuclei were pelleted, washed once with Micro-C buffer 3 (50mM Tris-HCl pH 7.5, 10mM MgCl2), and resuspended in 1x NEB T4 ligase buffer with 100μg/mL BSA. Each tube was treated with 10,000U of T4 DNA ligase (New England Biolabs #M0202) overnight at 25 °C to ligate spatially proximal fragments.

The next day, ligated nuclei were pelleted, resuspended in 1x NEBuffer 1 (New England Biolabs #B7001S; 10mM Bis-Tris-Propane-HCl, 10mM MgCl2, 1mM DTT), and treated with 2000U exonuclease III (NEB #M0206) for 15 minutes at 37 °C to remove biotin-dNTPs from any unligated fragment ends. Suspended nuclei were supplemented with 2mg/mL proteinase K (Viagen Biotech #501-PK), 0.1mg/mL RNase A (ThermoFisher #EN0531), 250mM NaCl, and 1% SDS (Sigma-Aldrich #L3771) and incubated at 65 °C overnight to reverse crosslinking.

The following day, the ligated DNA fragments were purified with a Zymo DNA Clean and Concentrator kit (e.g. Zymo Research #D4034) following kit instructions, save for the use of a larger elution volume of 50μL (2x25μL elutions per column). The resulting DNA was run on a 1% agarose gel at 120V for approximately 60 minutes. A band from approximately 250bp-400bp corresponding to dinucleosome-sized fragments was cut out and DNA extracted using a Zymo gel purification kit (e.g. Zymo Research #D4008), with a larger elution volume of 30μL (2x15μL elutions per column). For each tube, 30μL of T1 streptavidin beads (Invitrogen #65601) were washed in 1xTBW (1M NaCl, 5mM Tris-HCl pH 7.5, 0.5mM EDTA pH 8.0, 0.1% Tween-20), then resuspended in 2xBW (2M NaCl, 10mM Tris-HCl pH 7.5, 1mM EDTA pH 8.0). The dinucleosomal fragments, plus enough water to dilute the buffer to 1X concentration, were added to the beads and incubated at 25 °C overnight on a rotator.

DNA fragments were prepared for Illumina sequencing while still bound to beads using an NEBNext Ultra II library prep kit (NEB #E7645S) following manufacturer’s instructions. Prepared libraries on beads were amplified by PCR using Q5 DNA polymerase to generate sufficient input for target enrichment. The minimum number of cycles required to generate enough input (6 to 8, depending on the sample) were performed to minimize the presence of PCR duplicates. Amplified libraries were bound to NEBNext sample purification beads (NEB #E7104), washed twice with 80% ethanol, and eluted in water.

A capture panel comprising 80-mer biotinylated DNA oligonucleotides tiled across the region of interest (mm39: chr3:33,804,149-35,704,149) was obtained from Twist Biosciences. Target enrichment was carried out on amplified libraries using this panel in accordance with Twist’s standard Target Enrichment Protocol. In brief, libraries were dried by vacuum, resuspended with Twist Universal Blockers (Twist Bioscience #100578) and mouse Cot-1 DNA (Invitrogen #18440016), and added to a solution of Twist Hybridization Mix (#104178) and the capture panel. The libraries were hybridized to the probes overnight at 70 °C, then bound to streptavidin beads (Twist #100983). Captured libraries were washed in Twist wash buffers and PCR amplified on beads using Equinox library amplification mix (Twist #104178). 6 to 8 PCR cycles were performed. Amplified libraries were bound to DNA purification beads, washed twice with 80% ethanol, and eluted in water. The final captured libraries were sequenced on an Illumina platform. To increase the amount of data, target enrichment was conducted twice on the pre-capture library to generate two post-capture libraries; each post-capture library was sequenced separately. Reads from the two libraries from the same biological replicate were combined before analysis. Mapping statistics per sample can be found in Table S8.

### Computational analysis: tagmentation mapping

We used the pipeline published in (12) to map SB integrations. For experiments using the -161 kb launch pad we mapped to the mouse reference genome (Release M23 – mm10), for the -116 kb launch pad we mapped to mm10 with an *in silico* insertion of the SB cassette (including exact flanking indels from the CRISPR insertion) and lifted over the integration positions to the mm10 genome. In the pilot experiment (Fig. 1C), the pipeline was also used to assign integrations to the 129S1 or CAST allele based on SNPs in the mapping reads, using SNP annotation of CAST and 129S1 alleles from the Mouse Genomes Project (https://www.mousegenomes.org/publications/ (41, 42)). Reads were filtered for a minimal mapping quality of 10. For the sorted pools, integrations with at least two unique mapping reads were retained. To ensure that high reporter expression is due to location and not due to hopping-induced *Sox2::mCherry* deletions, the integrations in the in the P1 pools were further filtered for having at least one read mapping each from the 5′ ITR and 3’ ITR (reverse and forward read). For the large unsorted control pools we filtered integrations less stringently, including any integration with at least one mapping read. Note that the integrations in these large unsorted pools are not mapped exhaustively. In a few experiments, integrations exactly matching the -39 kb launch site (chr3:34721183-34721192) or an earlier derived hopped clone (chr3:25018987 and chr3:35232946) were found across many pools. This indicated a PCR contamination from an earlier experiment. Therefore in each experiment we filtered out all integrations exactly matching those coordinates or one of the other launch pads (-161 kb, -116 kb, +715 kb), with the exception of the launch pad that the reporter was mobilized from. The number of mapped integrations per cell line per population can be found in Table S7.

To identify the true integrations in clonal cell lines, we filtered more stringently, requiring at least ten unique mapping reads. Next, we discarded any locations that were supported by <20% of the read count of the top location, since these are likely to be either index-swapped reads from other clones or off-target reads. To call an insertion with confidence, we required to find exactly one integration supported by 5’ ITR and 3’ ITR mapping. For confident deletion clones, we required to find exactly two locations: one supported by the 5’ ITR mapping, and one by the 3’ ITR mapping. Any clones with more mapped locations were manually reviewed: any clones for which the extra reads could be explained by index-swapping were included, while clones with evidence of a secondary integration or an unexplained high number of mapping reads from a secondary location were discarded.

### Computational analysis: expression score

To calculate expression scores from sorted pools, we first combined the integrations from each experiment per sorted population. Identical integrations found in multiple sorted pools from one experiment most likely originated from a single hopping event. Still, we treated them as separate data points because they were independently sorted into an expression gate. Next, we estimated the fluorescence intensity of the hopped reporters in each expression gate (*Fluo*_*P*_) from the median fluorescence intensity as measured during the FACS sorting process (see Supplementary Text, **section “Expression score”**).

Between different experiments and populations, a different number of cells was sorted. These sorted cells (and thus the mapped integrations) also represent a different fraction of the unsorted cell population (see Supplementary Text, **section “Expression score”**). To account for this, we determined for each population *P* the correction factor *Fsort*_*P*_ (using a pseudo-count of 1 to account for sorted populations that were too rare to observe in the FACS recording):

FsortP=RecordedcellsinP+1TotalrecordedcellssortedcellsP

*F*_*sort*_ can be interpreted as the fraction of the original unsorted cell population that is represented by each sorted cell or mapped integration (under the assumption that each sorted integration is equally likely to be mapped). When for one cell line the number of replicate experiments was unequal between different populations, this was also accounted for in *Fsort*_*P*_ by multiplying by $\frac{\;minimum\;number\;of\;replicates\;}{\;number\;of\;replicates\;P}$

Next, for any given genomic window (W), the expression score was defined as the average of the population fluorescence (Fluo_P_), weighted by the number of integrations of each population in that window times the correction factor *Fsort*_*P*_.

$$\;expression\;scor{\text{e}}_{W}=\sum_{p=1}^{\text{n}}\;\left(\;Fsor{\text{t}}_{P}*N_{W,P}*\;Flu{\text{o}}_{P}\right)/\sum_{p=1}^{\text{n}}\;Fsor{\text{t}}_{P}*N_{W,P})$$

When pooling replicate data, *Fsort*_*P*_ and *Fluo*_*P*_ could vary between replicates. Therefore, the integrations were counted separately per replicate. When computing the Sox2P reporter expression score based on the three launch pads combined, their replicate experiments were combined in a similar way (6 replicates total: 2 replicates from -161kb, 3 replicates from -116kb [see Supplementary Text, **section “Expression score”**], 1 replicate from +715kb):

$$expression\;scor{\text{e}}_{W}=\sum_{E=1}^{\text{n}}\;\sum_{p=1}^{\text{n}}\;\left(\;Fsor{\text{t}}_{P,E}*N_{W,P,E}*\;Flu{\text{o}}_{P,E}\right)/\sum_{E=1}^{\text{n}}\;\sum_{p=1}^{\text{n}}\;\left(\;Fsor{\text{t}}_{P,E}*N_{W,P,E}\right)$$

To obtain a running window expression score (e.g. 10 kb window), integrations were counted in smaller bins (e.g. 1 kb, this is the step size) and the expression score was calculated from a group of bins (e.g. 10 bins). Window and step size were selected based on the density of integrations in each experiment and region of interest. To account for the deletion in the ΔCBS_*Sox2::mCherry* cell line, we shifted all integrations after the deletion by the width of the deletion, calculated the running mean expression score and then shifted the bins after the deletion back to actual their genomic position. To estimate a 95% confidence interval, the genome-wide list of integrations for each cell line was bootstrapped 5000 times and the expression score was calculated from each bootstrap. Reported are the median of these bootstrapped expression scores as well as the 2.5^th^ to 97.5^th^ percentile (95% confidence interval), for each window with at least 3 sorted integrations. To obtain the strand-specific expression score, only integrations on a specific strand were included in integration count of a window (*N*_*W, P, E*_).

### Computational analysis: flowcytometry

To show the exact FACS gating used to sort SB-transposase-transfected cells, we loaded the FACSDiva experiment file (.xml) and the raw fcs files in R using diva_to_gatingset from the CytoML package (43). Color density plots were created using ggcyto and geom_hex. The other color density plots (e.g. showing *Sox2::mCherry* and *Sox2::eGFP* negative populations after CRISPR treatment) were created from gated single cells exported from FlowJo. The threshold for GFP or mCherry negative cells was determined by the 99th percentile of the WT control, while the threshold for GFP or mCherry positive cells was 1.5 times this value.

To compare the *Sox2* and reporter expression levels between clones, the fluorescent values were corrected and normalized. First, the median fluorescence intensity (MFI) was calculated for each fluorophore and each clone. Next, the MFIs of a mESC control cell line lacking any fluorescent protein were subtracted from the respective MFIs of the other samples, to correct for autofluorescence. Subsequently, the corrected mCherry and mTurquoise2 values were normalized by the corrected eGFP value of the same sample, to adjust for any global effects on *Sox2* expression. Because there was variation in the absolute MFIs between measurement days, we finally divided the normalized fluorescence values by the respective value of the original -116kb_Sox2P cell line, measured on the same day. In this way, we obtained relative *Sox2::mCherry* and reporter (mTurquoise2) expression levels that could be compared between measurement days and experiments (Fig. 3C, Fig. 5B, Fig. 5G-H, Fig. S2G, Fig. S4I, Fig. S6I, Fig. S6M). To compare the expression of all fluorophores without adjusting for global changes in SOX2 level, we divided the autofluorescence-corrected MFI values by those of the -116kb_Sox2P cell line (‘relative expression, not normalized’, Fig. S4I bottom, Fig. S7). The measurements of the clones derived by SB hopping (insertions and deletions, e.g. Fig. 3C) contained no wild-type control, so we used the wild-type control from a different day with closely matching fluorescent values of the original -116kb_Sox2P cell line.

To compute the relative reporter and *Sox2::mCherry* expression of individual SB-transposase-transfected cells (Fig. 5E), the MFIs of the wild-type mESC sample were subtracted from the fluorescent values of each cell. Next, the corrected mCherry and mTurquoise2 values were normalized by the eGFP value of the same cell. Finally, all individual cell measures were scaled to the corrected, normalized MFIs of the -116kb Sox2P cell line to be able to combine the data from two measurement days (biological replicates). Only cells in P2-P4 were used because P5 and P6 contain primarily many non-mobilized reporters (so individual cells do not represent a unique integration location) and no cells were recorded in P1.

To determine the modes of the fluorescence density distribution in clones with a bimodal reporter expression pattern, we used the function getPeaks from the flowDensity package (44) on the bi-exponentially transformed data (as in the figures). We extracted at most two highest peaks and selected the peak with the highest expression value. This mTurquoise2 value was corrected and normalized to compare between experiments as previously explained for MFI values.

### Computational analysis: CTCF ChIP

Raw sequencing data were filtered (>=Q15) and adapters were trimmed using fastP (45). Sequences were mapped to mm10 reference genome using the BWA alignment tool (0.7.17-r1188) after indexing the mm10 reference genome. The coverage files (bigwig files) were generated with RPKM normalization using deepTools (version 3.0). Peak calling was performed with MACS2 at a q-value cutoff of 0.05 and CTCF motifs were called using Jaspar (CTCF:MA0139.1) database running MotifScan (v1.3.0). By manual inspection of the CTCF bigwig files we could see that one site was clearly bound but not annotated by the MotifScan. Therefore we added it manually (mm10; chr3: 34772210).

### Computational analysis: RNA stability and half-life

For each sample, the mean Ct value from technical triplicates was calculated and normalized to the corresponding T_0_ sample. ΔCt values were computed relative to T_0_, and relative RNA abundance was estimated as 2^(-ΔCt)^. Nonlinear least squares regression was used to fit a one-phase exponential decay model for each replicate: abundance = Y_0_ × exp(-*k* × t) using R. The decay constant *k* was extracted for each target, and RNA half-life was calculated as ln(2)/*k*. Fits were performed per replicate, per cell line, and target gene.

### Computational analysis: Estimation of RNA level reporter

The qPCR-primers used to measure GFP, mCherry and mTurq expression all have close to 100% efficiency (data not shown). Therefore, we estimated the relative expression of the reporter based on the T_0_-measurements from the RNA degradation experiment and RT-qPCR on one regular untreated RNA sample (n = 5), as follows. Per biological replicate, we calculated the mean ΔCt between the mTurq (reporter) and the two *Sox2* alleles (GFP and mCherry) as follows: ΔCt_mTurq = ((Ct_mTurq - Ct_GFP) + (Ct_mTurq - Ct_mCherry))/2. Thus the estimated relative level of mTurq is 2^(-ΔCt_mTurq)^ and the confidence interval is calculated as 2^(-ΔCt_mTurq ± 1.96×standard deviation).

### Computational analysis: region capture Micro-C of reporter cell lines

RCMC data analysis was performed in an allele-specific manner using custom 129S1/CAST F1-hybrid genomes. To construct the 129S1/CAST genome, mouse strain-specific variants were obtained from the Mouse Genomes Project (42), homozygous SNPs were filtered using SnpSift v4.3p (46)), and bcftools v1.9 (47) was used to insert single nucleotide variants into the GRCm38/mm10 mouse genome obtained from the Ensembl database. The resulting hybrid genome was further modified to generate custom genomes for each of the cell lines used in the RCMC (F121_Sox2_GFP/mCherry; LP-116 kb_Sox2P; LP-161 kb_Sox2P; LP-116 kb Sox2P_mChDel; LP-161 kb_Sox2P_mChDel; LP-161 kb_Sox2P_CDS_mChDel). To do this, the Sox2P or Sox2P-CDS inserts were added at -116 kb or -161 kb launch pad coordinates on the 129S1 allele. In order to unify the size of the chromosomes, the corresponding locus of the CAST allele was filled with N-stretches. If the editing was absent in the cell line or the *Sox2* gene deletion was present, the corresponding genomic regions of 129S1 allele were additionally modified with N-stretches. In addition, the mCherry and eGFP tag were added to the *Sox2* gene on the 129S1 and CAST allele respectively.

RCMC paired-end reads were aligned to the custom hybrid 129S1/CAST genomes using bwa-mem v0.7.17-r1188 (48) with the “-SP” options. The aligned reads were processed using pairtools v1.1.2 (49) following the haplotype-resolved guideline from the pairtools. First, the reads were parsed into contact pairs using pairtools parse with the parameters “--add-columns mapq,XA,NM,AS,XS”, “--walks-policy mask” and “--min-mapq 0”. Secondly, the pairs were phased using pairtools phase in “--tag-mode XA”. Finally, pairtools dedup was used with the parameters “--backend cython” and “--max-mismatch 1” to remove duplicates. The remaining reads were filtered to contain only pairs where at least one end was phased and both ends originated from the captured locus of interest, to avoid downstream biases described in the original RCMC study (18). These resulting reads were then converted to MCOOL format and iteratively corrected using cooler v0.10.3 (50).

Generated RCMC data has been deposited at the GEO database (GSE275427). Processing statistics of the RCMC data are provided in Table S8. Code for the RCMC analysis is available on Zenodo (zenodo.org/records/15681363) (51).

### Computational analysis: published Region capture Micro-C data

To visualize the processed RCMC data from (18), aligned to the mm39 genome, all annotations of the *Sox2* locus were lifted over to the mm39 genome (Fig. 1B and Fig. S6B). Insulation score was calculated using the GENOVA package (52) with the function insulation_score, on the 1 kb resolution data, with window = 20 (20 kb window size). To correlate contact probability to expression score, we used the GENOVA package to create a virtual 4C on the mm39 genome using as a viewpoint the region containing the two essential enhancer elements in the SCR (mm39: chr3:34811722-34816213, (16)) at 1 kb resolution. Then we lifted over the contact probabilities to the mm10 genome build and calculated the expression score on the same 1 kb bins. Only bins with at least 3 reporter integrations were included in the correlation. The correlation with genomic distance was calculated on the same bins, using the distance from the center of the bin to the center of the viewpoint.

### Computational analysis: transcription factor binding at the *Sox2*-CDS

To explore which transcription factors might bind at the *Sox2*-CDS, we downloaded the JASPAR CORE 2024 predicted transcription factor binding sites in the region around the *Sox2* gene from the UCSC track data hub (53) and filtered for a maximum p-value of 10^-3^ (score >=300). For all binding sites overlapping the *Sox2*-CDS we extracted the transcription factor. For compound motifs, both factors were extracted. Next, we searched the Cistrome Data Browser (54) for ChIP-seq datasets for these factors from mouse embryonic stem cells (filtering for the cell type “Embryonic Stem Cell”). All datasets with a called peak overlapping a corresponding predicted binding site on the CDS were selected. These datasets were manually filtered based on the information in the GEO accession display tool, to only include datasets from non-perturbed or control conditions, for the correct ChIP target. When multiple datasets remained for the same factor, we selected either replicate 1 (for ZIC2 and ZFP57) or the most recent dataset (for POU5F1). Next, the reprocessed bigwig tracks were downloaded from cistrome (http://dc2.cistrome.org//genome_browser/bw/X, where X is the cistrome ID) and visualized using the BigwigTrack function from Signac (55), smoothing over 100bp.

## Supplementary Material

Supplementary Information and Figures

## Data Availability

Laboratory notebooks and supplementary data (including plasmid sequences) are available on Zenodo (10.5281/zenodo.15674627). Raw sequencing data is available on GEO under accession numbers GSE275427. Code and supplementary files used as input for the scripts are available on Zenodo (zenodo.org/records/15681363, zenodo.org/records/15690525) (51, 56). All plasmids used will be made available upon request.
