# Supplementary material for: Functional maps of a genomic locus reveal confinement of an enhancer by its target gene
Source: Science. Author manuscript; Available in PMC 2025 Nov 17. (PMC7618358; doi:10.1126/science.ads6552)
Supplement: Supplementary Information and Figures [file EMS209699-supplement-Supplementary_Information_and_Figures.pdf]

Supplementary Materials for

Functional maps of a genomic locus reveal confinement of an enhancer by its target gene

Mathias Eder†, Christina J.I. Moene†, Lise Dauban, Mikhail Magnitov, Jamie Drayton, Marcel de Haas, Christ Leemans, Martijn Verkuilen, Elzo de Wit, Anders S. Hansen, Bas van Steensel*

†These authors contributed equally to this work.
*Corresponding author: b.v.steensel@nki.nl

The PDF file includes:

Supplementary Text
Figs. S1 to S8
Tables S1 to S7
References

Other Supplementary Materials for this manuscript include the following:

Table S8

Supplementary Text

Expression score

To correctly estimate the relative expression per location from the sorted integrations, we took several aspects of the reporter hopping data into account:

1. SB hopping is non-uniform, so the reporter integrations in the unsorted cell pool are not distributed equally across the locus.
2. Across the locus, the number of positions with a particular expression level is not equal: very high reporter expression might occur in fewer positions than low expression.
3. In a substantial fraction of the unsorted cells, the reporter is not mobilized and still in the launch pad. These cells all have the same reporter expression (+/- some noise), which overlaps with some of the sorting gates. As a result, the sorted populations that overlap with the launch pad expression level contain many unhopped integrations. This is why the number of mapped hopped integrations for those populations is lower than for other populations with a similar number of sorted populations (e.g. compare figure S2C, left panel, P2 vs P4). In addition, this means that the distribution of expression in the unsorted pool is not equal to the average of the distributions of expression of the *hopped* integrations.
4. Due to point 2 and 3, the different FACS sorting gates contain a different fraction of the unsorted population, with the most abundant gate containing around 5000 fold more cells than the least abundant gate (-116 kb Sox2P P1 vs P5, or -161 kb Sox2P and +715 kb Sox2P P2 vs P6). With the current mapping technology, it is not feasible to sort and map 5000 times as many integrations for these most abundant populations while preserving sufficient coverage of the lowly abundant populations. Therefore, the mapped integrations in the different populations reflect a different number of unsorted cells.

The reporter expression score was designed to take these aspects into account. In short, for each position in the locus we calculate the average of the reporter fluorescence values of the sorted populations, weighted by the number of mapped integrations per population. Furthermore, a few simple correction factors (see below and in methods) account for the unequal representation of the populations in the sorted data (points 2-4 above). The non-uniform hopping distribution (point 1) is automatically accounted for, because all populations in one experiment are sorted from the same hopping distribution. Therefore, positions with more integrations will be proportionally more abundant in each of the expression gates and the hopping distribution will not affect the estimated expression score. However, regions with more integrations in the unsorted pool will contain more data to base the expression score on, thus resulting in a smaller confidence interval of the expression score (based on bootstrapping, see methods).

To estimate the fluorescence intensity of the hopped reporters in each expression gate ($Fluo_P$), we generally take the median fluorescence intensity (MFI) of each sorted population, as measured during the FACS sorting process. However, when a gate contains many ‘unhopped’ cells (i.e. the gates overlapping the expression range of the launch pad reporter), the MFI of the gate does not accurately represent the hopped reporters. For the three reporter cell lines with a Sox2P-reporter and Sox2::mCherry present (-161 kb Sox2P, -116 kb Sox2P, and +715 kb

Sox2P) we therefore obtained a shared estimated $Fluo_P$, by averaging for each population the MFI of those cell lines not overlapping the launch pad expression reporters (i.e. P1 from -116 kb and +715 kb, P2-P5 from -161 kb and +715 kb, P6 from -116 kb), across the replicates. Note that for -161 kb there were P1 cells sorted, but no cells were recorded (frequency was less than 1 in 100,000 cells). For each of the other two hopping experiments (Sox2P ΔCBS_Sox2::mCherry and Sox2P_CDS) we only used one launch pad cell line, so we used the MFI per gate and per replicate of that cell line as $Fluo_P$, even when the gate overlapped the launch pad expression.

While in most of the experiments we used the same six populations (P1-P6), the expression score does not require equally sized gates. As long as the gates cover the entire expression range of the unsorted cell pool, any change in the sorting strategy will be accounted for by the corresponding changes in $Fsort_P$ and $Fluo_P$. This allowed us to use the split gates P1 and P2 into P1-H, P1-L, P2-H and P2-L to further refine the expression pattern in -161 kb Sox2P ΔCBS_Sox2::mCherry. However, in two cases a part of the expression range of the unhopped reporters was covered by more replicates than the rest of the range: for -116kb Sox2P (Sox2::mCherry intact) 50,000 cell pools (replicate 4) were sorted for P3-P5, but not for P1, P2 and P6; in replicate 4 of -161 kb Sox2P ΔCBS_Sox2::mCherry we sorted both a 50,000 cell pool for P2 as well as 1000 cell pools for P2-H and P2-L (covering the same expression range). To account for this, we corrected $Fsort_{P,E}$ of those populations with more replicates with the factor $\frac{minimum\ number\ of\ replicates}{number\ of\ replicates\ P}$ (0.75 and 0.8, respectively).

Bimodality of reporter expression upon *Sox2* deletion

Although deletion of *Sox2::mCherry* can cause substantial upregulation of reporter expression, we noticed that in the -116 kb and -39 kb reporter cell lines, a sub-population of cells lost reporter expression upon this deletion **(Fig. 4B)**. The same loss of reporter expression occurred in a subset of cells with a *Sox2::eGFP* deletion (**Fig. S4A**). In addition, at the -116 kb location the control transfections with a single gRNA upstream of the *Sox2* gene, also caused loss of reporter expression in some cells (**Fig. S4A**, up1c_single, up2c_single). These transfections with a single gRNA do not cause deletion of the full *Sox2* gene (because the cells are still positive for *Sox2::eGFP* and *Sox2::mCherry*). However, they may cause mutations or deletions at the cut site due to imperfect repair. Specifically the two upstream gRNAs, but not the downstream gRNA or control gRNA (cutting on a different chromosome), can cause downregulation of the -116 kb reporter, which is only a few kb away from the upstream cut sites. Therefore, we suggest that this is a direct effect of cutting on the 129S1 allele (containing the reporter) causing damage to the reporter. If single gRNA cutting can cause silencing of the -116 kb reporter without a loss of *Sox2* expression, this might also explain the loss of reporter expression in the *Sox2::eGFP*-negative cells. Besides a complete deletion of the *Sox2::eGFP*-allele, these cells can also have a single upstream cut on the 129S1 allele (containing reporter and *Sox2::mCherry*) that caused the loss of reporter expression.

The cells losing reporter expression at the -39 kb location cannot be explained by the same mechanism, since this reporter is not close to any of the gRNA cut sites. Indeed, for this reporter silencing only occurs upon loss of a *Sox2* allele, and not in any of the single gRNA transfections. Therefore, it is possible that this silencing is a *trans*-effect of the 50% loss of SOX2 protein level

due to deletion of one *Sox2* copy. We note that the expression of *Sox2::mCherry* is still normal in the *Sox2::eGFP* deleted cells, indicating that the reduced SOX2 level does not cause differentiation of the cells (**Fig. S4B**). However, we have noted across experiments that the -39 kb reporter location is less stable than the other cell lines: a small subset of cells spontaneously acquire reduced reporter expression (see the lower tail in the untreated cells in **Fig. S4A**) and when we insert reporters in the launch pad the clone-to-clone variation is relatively large (**Fig. 5B**, right panel). It is therefore possible that the reporter at position -39 kb is more sensitive to a reduction in SOX2 protein level than the promoter of the endogenous *Sox2* gene, which is in an optimal location and is boosted by the *Sox2* CDS.

Finally, we tested whether the effect of *Sox2::mCherry* deletion is stable over time, by sorting pools and clones of *Sox2::mCherry* negative cells with high reporter expression. In the -161 kb and -39 kb location, expression remained stable and uniform **(Fig. S4C-E)**. In contrast, at -116 kb, a fraction of cells reproducibly lost reporter expression in both sorted pools and clones **(Fig. S4C, S4F-G).** Possibly, the H3K27me3-rich region just upstream may spread into the -116 kb insertion site upon loss of active chromatin at the endogenous *Sox2* locus **(Fig. S4H)**. Such epigenetic silencing might also explain the few clones with hopping-induced *Sox2::mCherry* deletions that exhibit bimodal reporter expression (**Fig. S3C**), since these reporters are flanked by the same upstream sequence of the -116 kb launch pad.

Despite these various unexpected causes for reporter silencing (direct damage by gRNA cutting, *trans*-effects of SOX2 level, epigenetic silencing), in each location reporter upregulation is specific to the deletion of *Sox2::mCherry*, indicating that this is a bona fide *cis*-effect of the gene deletion.

Eder, Moene et al. Fig S1

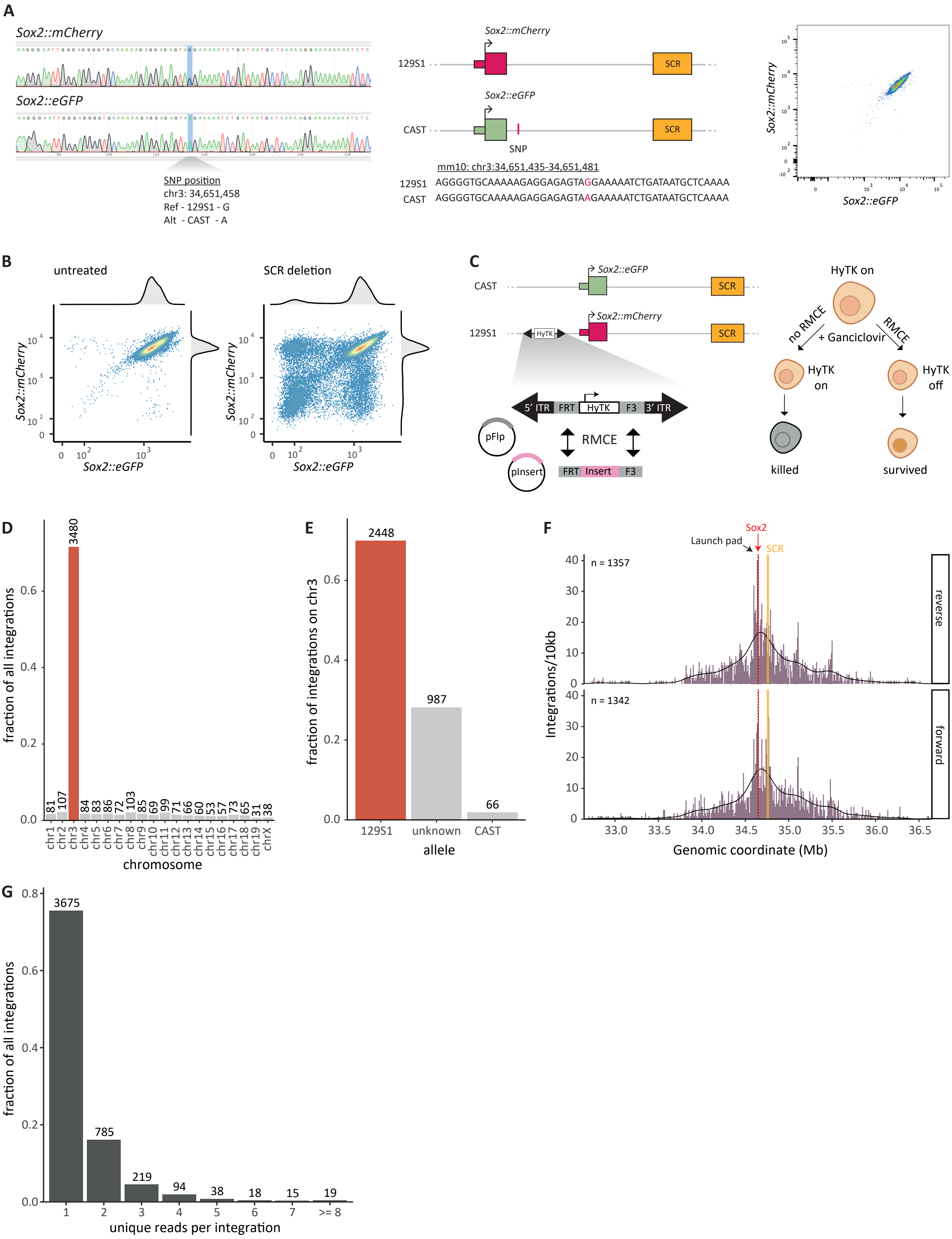

Fig. S1. Cell line generation and proof-of-principle experiment

(A) Left and middle: Allele specific genotyping of *Sox2::mCherry* and *Sox2::eGFP* by PCR and sanger sequencing. Right: *Sox2* allelic expression levels measured by flow cytometry. **(B)** Effect of CRISPR/Cas9 mediated SCR deletion on *Sox2::eGFP* & *Sox2::mCherry* expression in bulk. Left: untreated control, right: gRNAs against SCR. **(C)** Recombination-mediated cassette exchange: The hygromycin phosphotransferase-thymidine kinase fusion gene (HyTK) is flanked by heterotypic Flp recombinase recognition sites (FRT & F3) and embedded into a Sleeping Beauty transposon (SB-ITRs), integrated via CRISPR/Cas9 mediated knock-in 6 kb upstream of the *Sox2::mCherry* fusion gene. Co-transfection of the Flp recombinase expression plasmid (pFlp) and donor plasmid (pInsert) with subsequent ganciclovir selection for 7 days results in cells with the intended insert. **(D-G)** Statistics from the proof-of-principle hopping experiment, where a SB transposon carrying a 282 bp random DNA sequence was relocated. Number of integrations is indicated (above the bars or n). **(D)** Fraction of integrations mapped per chromosome. Launch pad is on chr3. **(E)** Fraction of all integrations on chr3 mapped to each allele, or not assigned to either allele. The launch pad is on the 129S1 allele. **(F)** Number of integrations per 10 kb bin, split by SB orientation. **(G)** Distribution of the number of unique mapping reads supporting each integration. (B, D-G) Data from one (proof-of-principle) biological replicate.

Eder, Moene et al. Figure S2

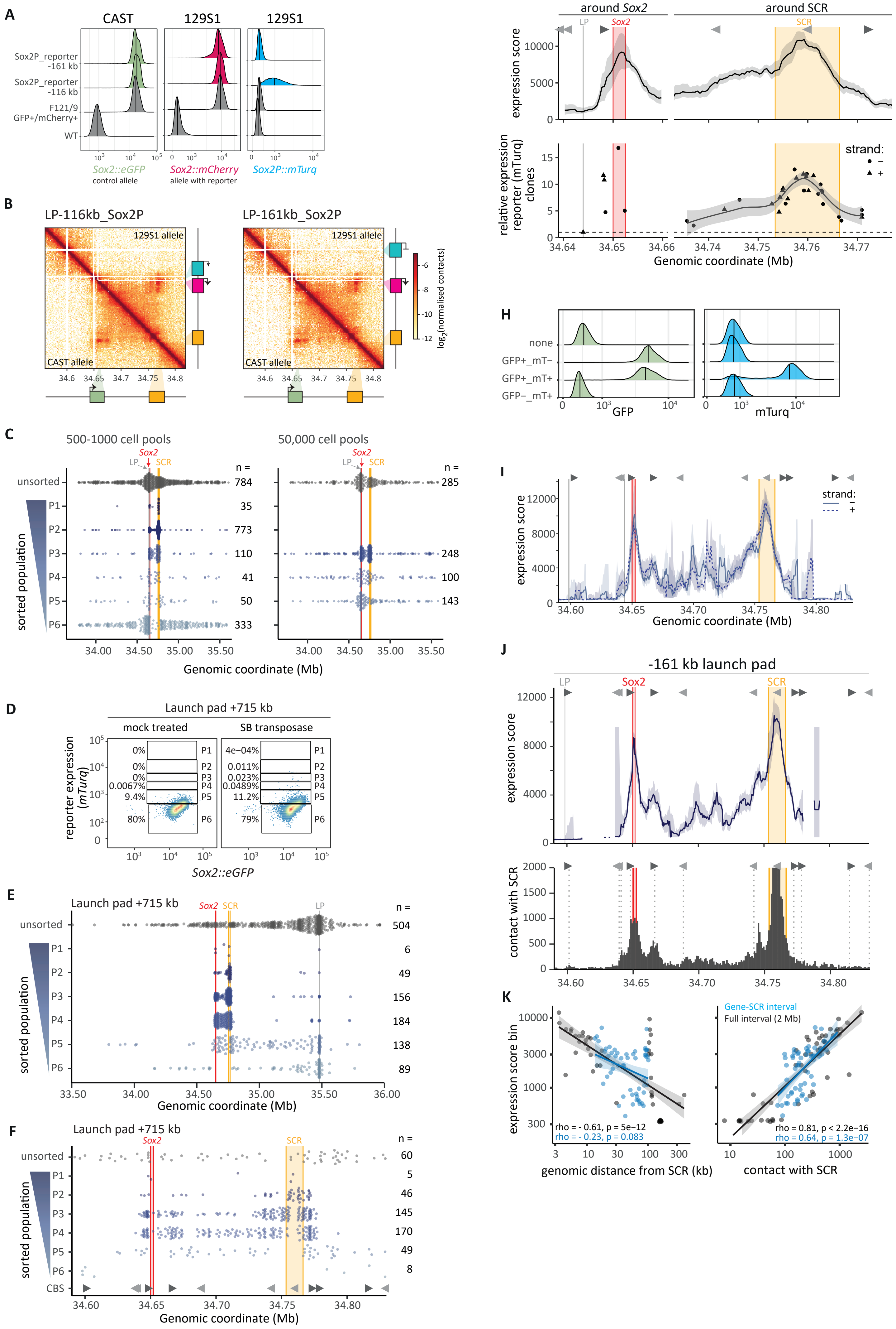

Fig. S2. Reproducibility of reporter hopping and validation of the expression score.

(A) Flow cytometry profiles showing expression of *Sox2::eGFP* (green), *Sox2::mCherry* (red), and Sox2P reporter (mTurq, blue) in cell lines with reporters at -161 kb or -116 kb, compared to the parental cell line with fluorescently tagged *Sox2* (F121/9 GFP+/mCh+) and wildtype mESCs. **(B)** Allele-specific region capture Micro-C (RCMC) contact maps of the Sox2 locus in LP-116 kb_Sox2P (left) and LP-161 kb_Sox2P (right) cell lines. Top-right triangles show contact frequencies of the 129S1 (reporter-containing) allele and bottom-left triangles show contact frequencies of the CAST (control) allele. Two biological replicates combined per cell line. Same data as **Fig. S5B-C**. **(C)** Mapped integrations from unsorted and sorted cell populations (P1–P6) mobilized from the -116 kb launch pad. Each dot indicates one integration. Left: combined data from three replicates with 500–1,000 cells per sorted pool. Right: single replicate of one 50,000-cell pool each sorted for P3–P5. **(D)** Reporter (mTurq) and *Sox2::eGFP* expression profiles in mock-treated and SB-transposase transfected cells containing the +715 kb launch pad, measured by FACS. Percentages indicate the fraction of cells in each gate (P1–P6). **(E)** Mapped integrations from a large pool of unsorted cells (ctrl) and the six sorted populations (P1-P6), mobilized from the +715 kb launch pad. Each dot indicates one mapped integration, number of plotted integrations in each population is indicated on the right. Single biological replicate. **(F)** Zoom-in of (E) with annotated CTCF binding sites (CBS; triangles indicate orientation). **(G)** Top: expression scores around *Sox2* gene and SCR based on the sorted integrations (also shown in **Fig. 2H**), smoothened using a 5 kb running window shifted in 500 bp steps. Bottom: relative reporter expression (median mTurq, estimated autofluorescence subtracted, normalized to eGFP and to the reference -116 kb integration) of clones around the *Sox2* gene and the SCR. Loess curve shows the trend in expression. Flow cytometry measurements of clones have no spectral spillover (see H). **(H)** Control for mTurq-to-GFP spectral spillover in analytical FACS. No spillover was detected in single-color clones. **(I)** Strand-specific expression scores (forward and reverse orientation), across the *Sox2* locus, from all three launch pads combined (LP-161, LP-116, LP+715). Smoothened using a 5 kb running window, shifted in 500 bp steps. Shaded region indicates 95% confidence interval. **(J)** Top: Expression score derived from LP-161 kb (data shown in **Fig. 2G**), smoothened using a 5 kb window shifted in 500 bp steps and only plotted when window contains 3 or more SB integrations. Bottom: virtual 4C contact profile (from RCMC data, (18)) with the center of the SCR as viewpoint, at 1 kb resolution. Dashed lines indicate CBSs and grey triangles indicate their orientation. **(K)** Correlation between expression score of LP-161 kb (J, top) and genomic distance to the SCR (left) or virtual 4C contact with the core SCR (right), per 1 kb bin on the ~2Mb genomic *Sox2* region (interval in black) and the *Sox2*-SCR interval (5 kb after the end of the gene till 5 kb before the SCR) in blue. Only bins with 3 or more SB integrations are included. Rho is Spearman's correlation coefficient.

Eder, Moene et al. Figure S3

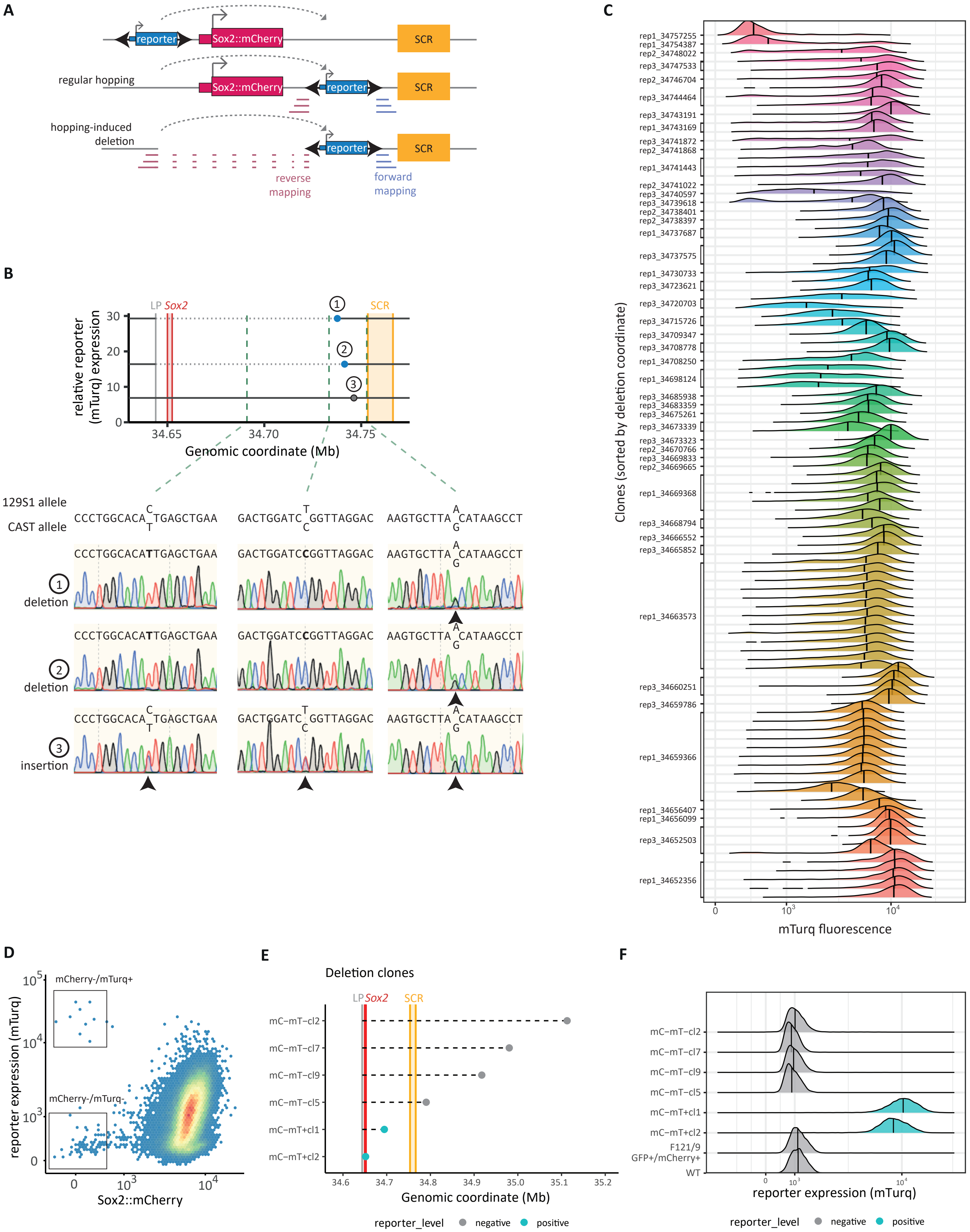

Fig. S3. Mapping of hopping induced deletions and insertions.

(A) Schematic of Tn5-based mapping in cells with a regular hopping (top) versus hopping-induced deletions (bottom). In deletion events, one SB inverted terminal repeat (ITR) maps to the original launch site and the other to the insertion site. **(B)** Confirmation of the loss of the 129S1 allele in the deleted region of deletion clones. Top: genomic location and expression of two hopping-induced deletion clones (1, 2, blue) and one regular insertion clone (3, grey). Horizontal dashed grey lines indicate expected deletions, vertical dashed green lines indicate the locations of three known SNPs between the 129S1 and CAST alleles. Bottom: Sanger sequencing across the three SNPs in the three cell lines. Black arrowheads indicate double peaks at the SNPs, indicating that both alleles are present. When only the CAST allele is present, the SNP is indicated in bold in the sequence above the Sanger trace. **(C)** Reporter (mTurq) fluorescence of individual deletion clones (shown in **Fig. 3B**), ordered by deletion-end coordinate. For some deletions, multiple clones were derived and measured (probably subclones from the same hopping event). Data from three measurement days are shown together without normalization, replicates are indicated per insertion (rep1-3). **(D)** Illustration of approximate gating strategy used to isolate clones lacking *Sox2::mCherry* with or without reporter expression (top and bottom rectangles, respectively). **(E)** Genomic coordinates of deletion clones with (blue) or without (grey) reporter expression, mapped along the *Sox2*-SCR region. Vertical bars mark *Sox2* (red) and SCR (yellow). **(F)** Reporter expression profiles of clones shown in (E).

Eder, Moene et al. Figure S4

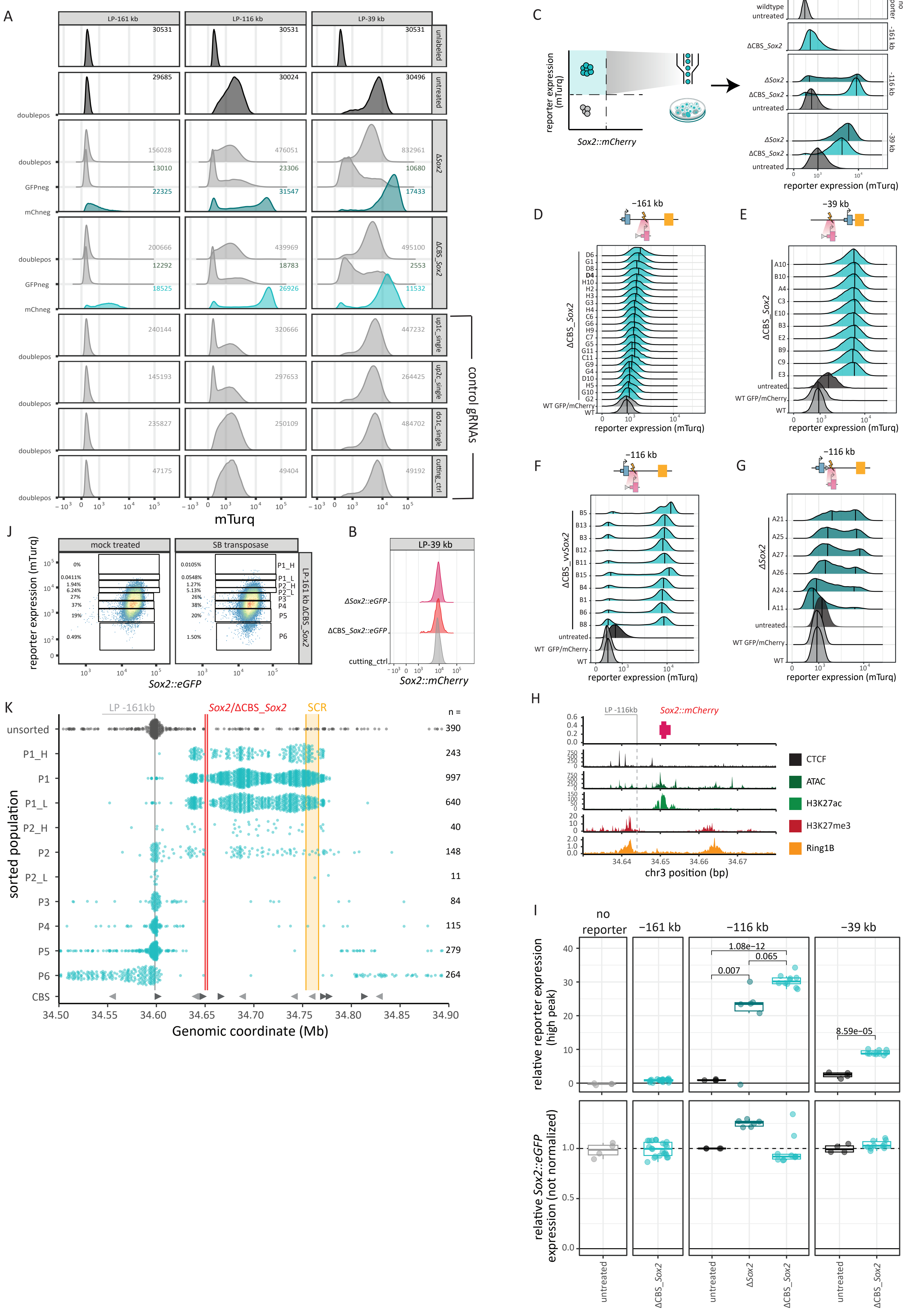

Fig. S4. Sox2P-reporter expression in *Sox2::mCherry* deleted pools and clones.

(A) Reporter (mTurq) expression across three launch pad positions (-161 kb, -116 kb, -39 kb), with or without *Sox2::mCherry* (or CBS_*Sox2::mCherry*) deletion. Controls include single gRNA transfections (up1c, up2c, do1c, cutting control). Reduced reporter expression upon up1c and up2c transfection at -116 kb suggests gRNA-specific, not deletion-specific, effects. **(B)** *Sox2::mCherry* expression in *Sox2::eGFP*-negative LP-39 kb cells. *Sox2::mCherry* expression is similar to control. **(C)** Re-measured distribution of reporter expression in *Sox2::mCherry* negative, reporter-high sorted cell pools, cultured at least 12 days after sorting. **(D-G)** Reporter expression distributions of independently genotyped Δ*Sox2::mCherry* or ΔCBS_*Sox2::mCherry* clonal cell lines. **(D)** ΔCBS_*Sox2::mCherry* clones with reporter at -161 kb, clone D4 (bold) was used for hopping in Fig. 4C. **(E)** As in (D), for -39 kb reporter. **(F)** As in (D), for the -116 kb reporter cell line. **(G)** As in (D), for *Sox2::mCherry* deletion in the -116 kb reporter cell line. Untreated clones are shown in black. Vertical lines indicate the (at most) two highest peaks in the density distribution, the peak with the highest mTurq level is used in (I). **(H)** ChIP-seq tracks of CTCF (own data), ATAC-seq (20), H3K27ac (21), H3K27me3 (*57*) and Ring1B (17), smoothened using a 100 bp running window. Red indicates *Sox2::mCherry*; grey dashed line indicates reporter insertion at LP-116 kb. **(I)** Top: maximum reporter expression in clones from (D-G), autofluorescence-subtracted, normalized to *Sox2::eGFP* and the median of the control -116 kb reporter line. Dashed line = 1 (reference). Each dot is one clone (edited) or one measurement replicate on a separate day (untreated). Bottom: median *Sox2::eGFP* expression (autofluorescence-subtracted, relative to the median of the control -116 kb reporter line) in the same lines. **(J)** Reporter and *Sox2::eGFP* expression in untreated and transposase-transfected ΔCBS_*Sox2::mCherry* cells (LP-161 kb). Sorting gates P1_H-P6 shown for *Sox2::mCherry* negative single cells. Gating is representative of 2 replicates. **(K)** Mapped integrations from P1_H-P6 sorted populations after hopping from LP-161 kb in ΔCBS_*Sox2::mCherry* cells. Combined data from 5 replicates. Each dot indicates one integration; number of plotted integrations (n) shown on the right.

Eder, Moene et al. Figure S5

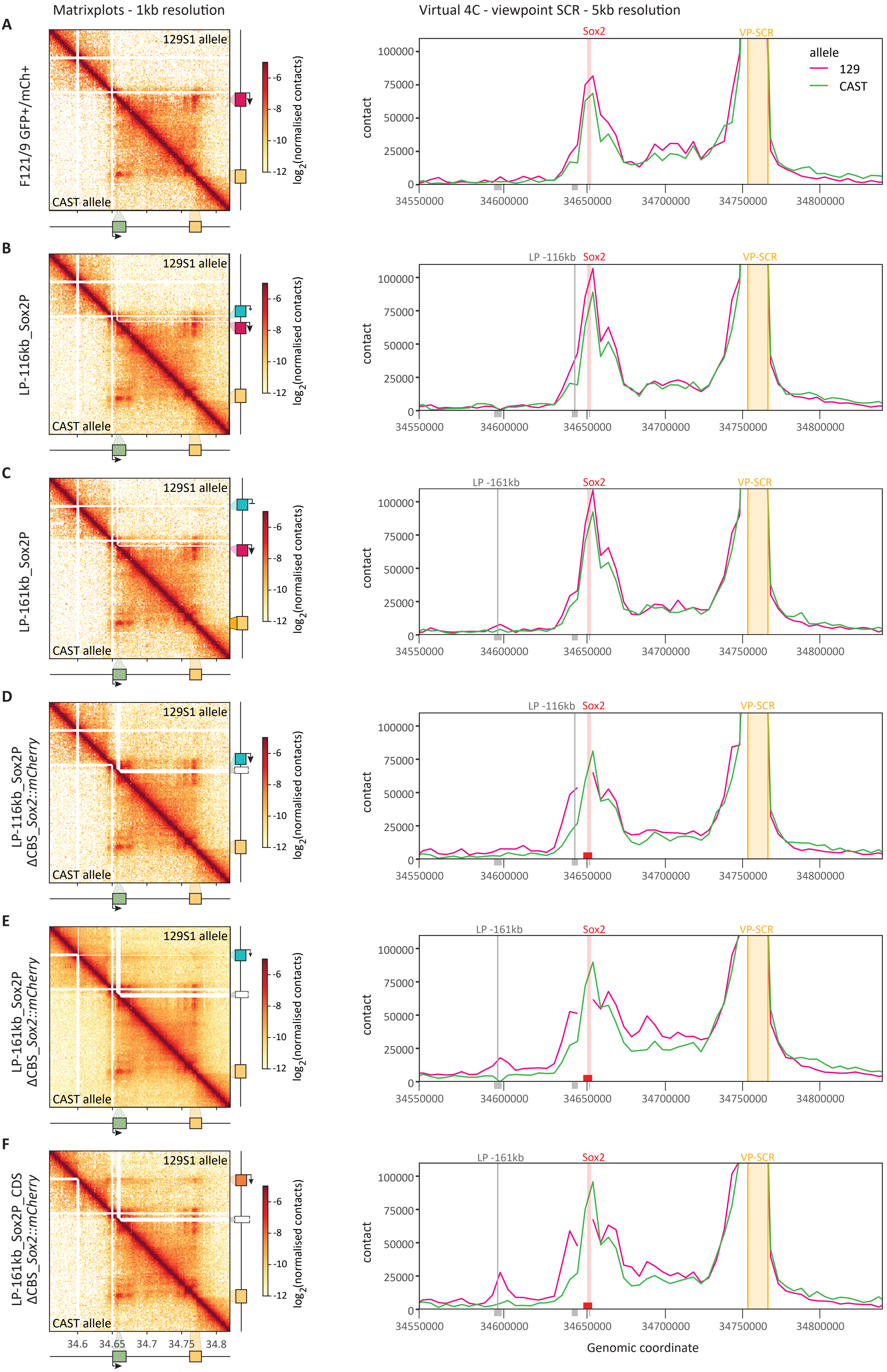

Fig. S5. Allele specific RCMC contact maps and virtual 4C of reporter cell lines in the presence and absence of the endogenous *Sox2::mCherry* gene.

(A-F) Left: Region capture Micro-C (RCMC) contact matrices (1 kb resolution) around the *Sox2*-SCR locus. Top-right and bottom-left triangles show allele-specific contact maps for the 129S1 (reporter-containing) and CAST (control) alleles, respectively. To the bottom and right of each matrix are schematic representations of the corresponding alleles, not to scale (red = *Sox2::mCherry*, green = *Sox2::eGFP*, yellow = SCR, blue = Sox2P reporter, orange = Sox2P_CDS reporter). Right: Virtual 4C plots (5 kb resolution) using the SCR as viewpoint, showing allele-specific contact profiles from the same datasets. Cell lines shown are:
(A) Double tagged control (*Sox2::mCherry* + *Sox2::eGFP*) without reporter integration,
(B) LP-116 kb_Sox2P
(C) LP-161 kb_Sox2P
(D) LP-116 kb_Sox2P with ΔCBS_*Sox2::mCherry* deletion (also called LP-116 kb_Sox2P mChDel)
(E) LP-161 kb_Sox2P with ΔCBS_*Sox2::mCherry* deletion (also called LP-161 kb_Sox2P_mChDel), and
(F) LP-161 kb_Sox2P_CDS with ΔCBS_*Sox2::mCherry* deletion (also called LP-161 kb_Sox2P_CDS_mChDel)
Reads were mapped to a custom genome assembly incorporating all possible genetic modifications (e.g., *Sox2::eGFP*, *Sox2::mCherry*, reporter integrations) and absent features (the LP not present, or ΔCBS_*Sox2::mCherry*) were masked per cell line. White stripes in matrix plots indicate non-mappable or masked regions. In the virtual 4C plots, genomic coordinates correspond to the mm10 genome, grey boxes denote insertions made in the custom genome (landing pads and fluorescent tag of *Sox2*) and red boxes (D-F) mark the genomic deletion of CBS_*Sox2::mCherry* on the 129S1 allele. Genomic coordinates in the contact matrices correspond to the modified genome.

Eder, Moene et al. Fig S6

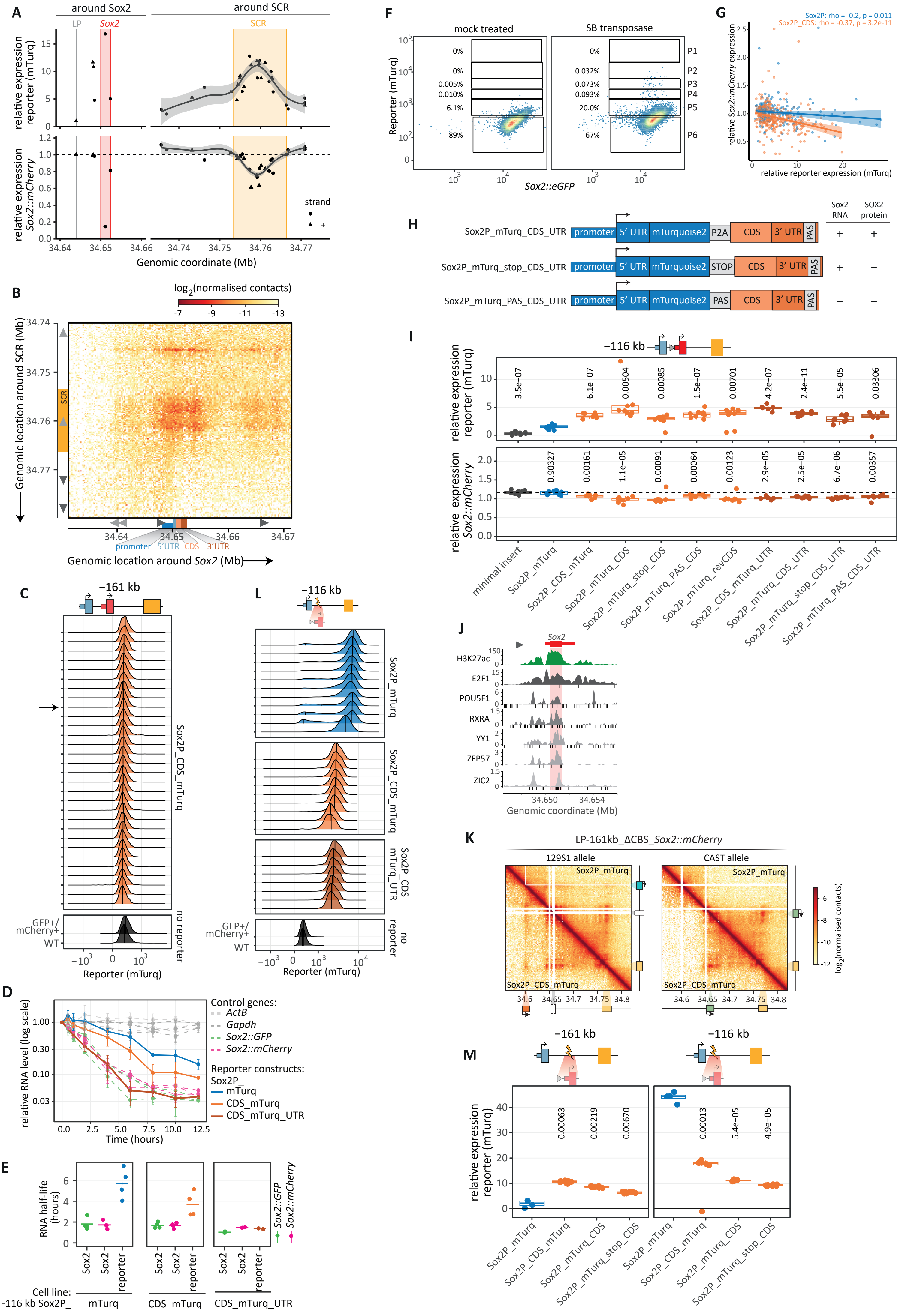

Fig. S6. Impact of the *Sox2* coding sequence on reporter and *Sox2* expression.

(A) Relative reporter (mTurq) and *Sox2::mCherry* expression in clones around *Sox2* and the SCR (median expression, estimated autofluorescence subtracted, normalized to GFP, and then normalized to the unhopped reporters at -116 kb). Loess curve shows the trend in expression. Same clones and mTurq data as in **Fig. S2G**, bottom. **(B)** Contact frequency between *Sox2* and the SCR (region-capture micro-C data from (18), 200-bp resolution). Positions of the components of the *Sox2* gene (blue, orange, red; x-axis) and SCR (yellow; y-axis) are indicated. Grey triangles indicate CTCF binding sites and their orientation. Arrows indicates the genomic direction. **(C)** Reporter expression levels in a panel of clones with the Sox2P_CDS reporter integrated at the -161 kb launch pad with *Sox2::mCherry* intact. The arrow indicates the clone used for hopping (see **Fig. 5C**). **(D)** RNA degradation time course of control genes (*ActinB, Gapdh*) and transcripts of interest (*eGFP, mCherry, mTurq*) in three different reporter cell lines (LP-116kb_Sox2P, LP-116kb_Sox2P_CDS & LP-116kb_Sox2P_CDS_UTR) measured by RT-qPCR. Cells were treated with 10 µg/ml ActinomycinD and harvested at t = 0, 0.5, 1, 2, 4, 6, 8, 10, 12 hours from treatment start. RNA was extracted and each qPCR was performed in technical triplicates. Shown are mean relative abundance ± standard deviation, from four biological replicates for Sox2P & Sox2P_CDS timepoints t = 0, 1, 2, 4, 6, two biological replicates for Sox2P & Sox2P_CDS timepoints t = 0.5, 8, 10, 12 and Sox2P_CDS_UTR t = 0, 1, 2, 4, 6, 8, 10, 12. **(E)** Estimated RNA half-lives (hours) from fitting an exponential decay model to data shown in (D), per biological replicate (dots). Horizontal line indicates the median. **(F)** Expression of *Sox2::eGFP* (control) and *Sox2* reporter (mTurq) in the untreated cells and cells transfected with the SB transposase plasmid, for the -161 kb Sox2P_CDS reporter cell line. The percentage of cells in each sorted gate is indicated. Cells are gated for *Sox2::mCherry*-positive single cells. Representative image of 2 biological replicates. **(G)** Correlation between relative *Sox2::mCherry* and reporter (mTurq) expression from the Sox2P and Sox2P_CDS reporter, from cells with reporter expression in the gates P2-P4 after induction of hopping. Same as **Fig. 5E**, but also showing the outlier points. **(H)** Design of reorganized CDS reporter (as in **Fig. 5F**) with endogenous 3'UTR instead of the SV40 polyA-signal. **(I)** Reporter and *Sox2::mCherry* expression for reorganized CDS constructs including 3'UTR containing constructs, as in **Fig. 5G**. All data except UTR-containing reporters are also shown in **Fig. 5G**. See **Fig. S7B** for raw expression values (not normalized to eGFP). Statistical significance calculated using Welch's t-test; for reporter expression every construct is compared to Sox2P, for *Sox2::mCherry* expression to 'minimal insert'. **(J)** ChIP-seq tracks of H3K27ac (*21*) and transcription factors (*58-63*) showing a peak overlapping a motif in the CDS (FIMO cutoff p-value < 10^{-3}). Called motifs are indicated by vertical black ticks below each track. Red indicates Sox2; thick red rectangle indicates Sox2 CDS; grey triangle indicates CTCF binding site. **(K)** Allele-specific contact maps (of the *Sox2*-SCR region comparing the reporter with (bottom triangle) or without the CDS (top triangle) in the ΔCBS_*Sox2::mCherry* background. Same data as **Fig S5C,E**. **(L)** Distribution of mTurq expression of reporters integrated at -116 kb in a ΔCBS_*Sox2::mCherry* cell line. Each density plot is one clone, vertical lines indicate the (at most) two highest peaks in the density distribution. **(M)** As (I) top, but in two LP positions (LP-161 kb, LP-116 kb) and in a ΔCBS_*Sox2::mCherry* deletion background.

Eder, Moene et al. Fig S7

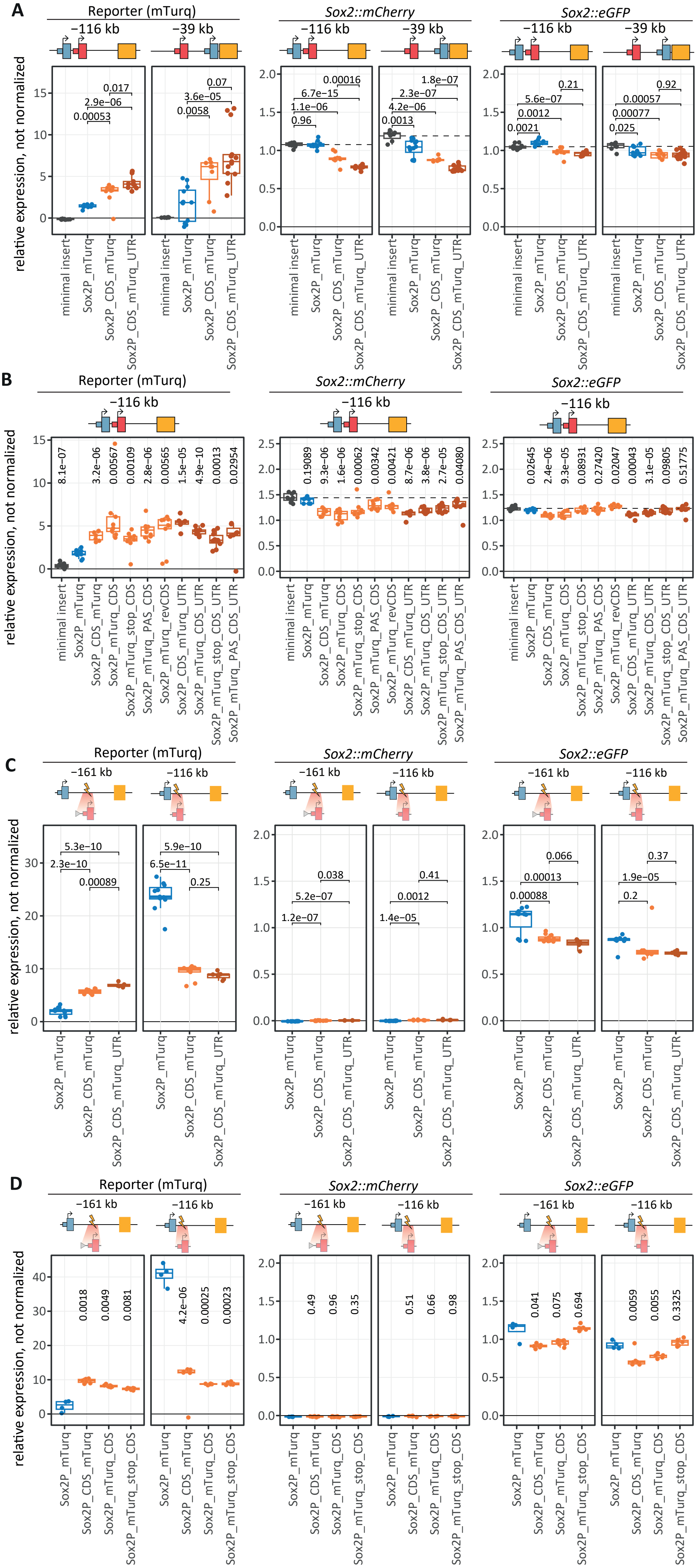

Fig. S7. Relative reporter and endogenous *Sox2* expression levels in different genomic locations and backgrounds.

(A) Relative expression of the reporter (mTurq), *Sox2::mCherry* and *Sox2::eGFP*, not normalized to *Sox2::eGFP*, for the three reporters inserted at LP-161 kb and LP-116 kb. Each dot represents one clone. Expression values are autofluorescence subtracted and normalized to the control reporter cell line (Sox2P reporter at LP-116 kb) measured on the same day. Dashed line on *Sox2::mCherry* and *Sox2::eGFP* indicate the median of the minimal insert. P-values according to Welch's t-test. **(B)** As in (A), but for original (Sox2P_mTurq, Sox2P_CDS_mTurq, Sox2P_CDS_mTurq_UTR) plus newly designed reporters inserted at LP-116 kb. Statistical significance calculated using Welch's t-test; for reporter expression every construct is compared to Sox2P, for *Sox2::mCherry* and *Sox2::eGFP* every construct is compared to 'minimal insert'. **(C)** As in (A), but for three reporters inserted at LP-161 kb and LP-116 kb in a ΔCBS_*Sox2::mCherry* deletion background. **(D)** Same as in (C), but including newly designed reporter constructs. Statistical significance calculated using Welch's t-test, comparing each construct to Sox2P.

Eder, Moene et al. Figure S8

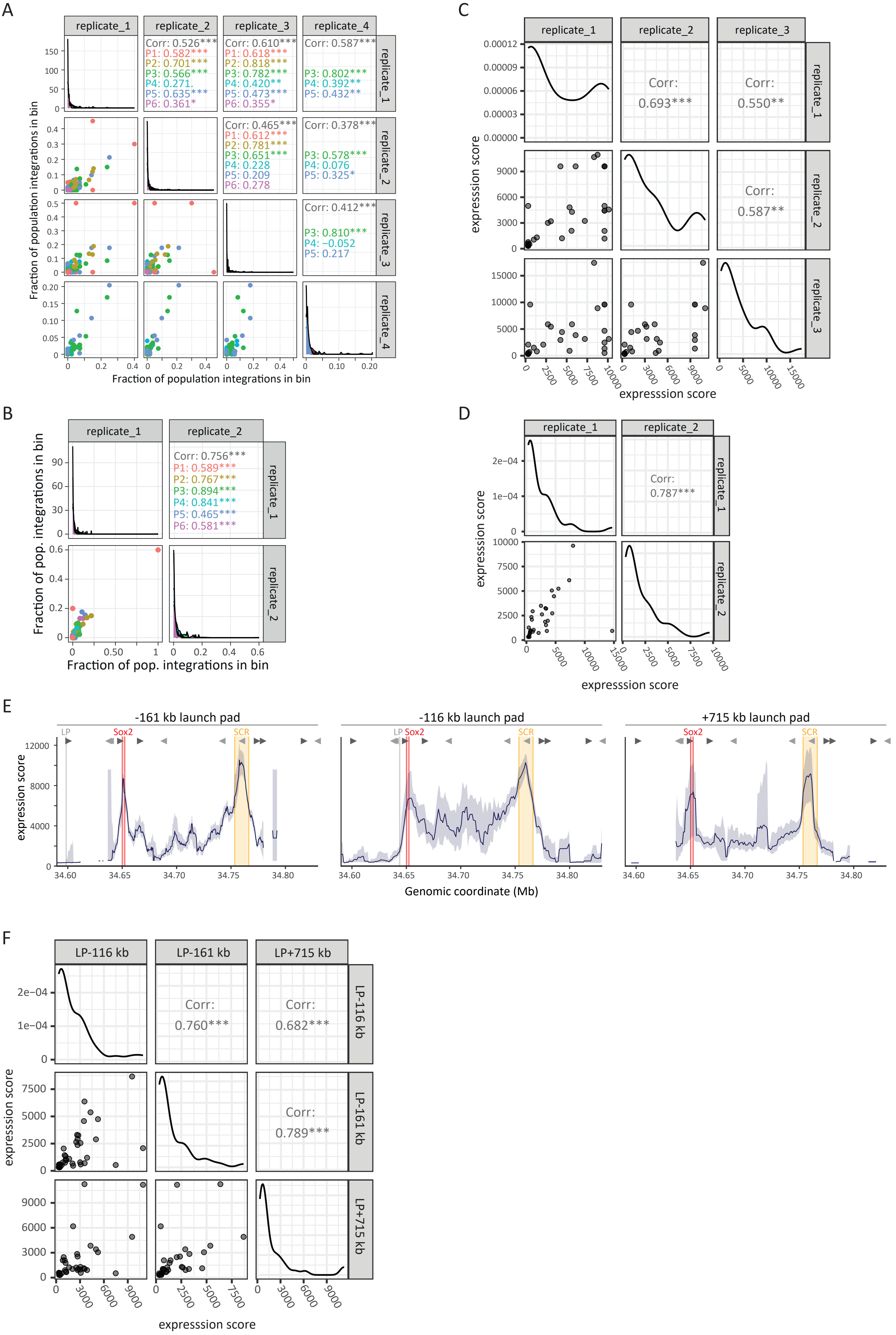

Fig. S8. Reproducibility of intra and inter launch pad specific hopping.

(A) Reproducibility of reporter integrations across the *Sox2* locus between biological replicates. Scatterplots show the fraction of population integrations per 5 kb bin (on the interval in **Fig. 2D**), colored by sorted population, across four replicates from the -116 kb launch pad. Diagonal panels show distributions per replicate; upper panels show Spearman correlation coefficients. **(B)** As in (A), for the -161 kb launch pad (two biological replicates). **(C)** Reproducibility of the expression score across the *Sox2* locus between biological replicates. Scatterplots show the expression score per 5 kb bin (on the interval in **Fig. 2D**) across three replicates from the -116 kb launch pad; upper panels show Spearman correlation coefficients. Expression score cannot be computed from replicate 4 alone, because it only covers populations P3-P5. **(D)** As in (C), for the -161 kb launch pad (two biological replicates). **(E)** Reporter expression scores derived from each launch pad: LP-161 kb, LP-116 kb, and LP+715 kb. LP -116 kb and +715 kb are smoothened using a 10 kb window shifted in 1 kb steps, LP -161 kb is smoothened using a 5 kb window shifted in 1 kb steps. Shaded region indicates 95% confidence interval and the expression score is only plotted when the window contains 3 or more SB integrations. LP-161 kb is also shown in **Fig S2J**. **(F)** As in (C), but comparing between the datasets from the three separate launch pads. For each launch pad all replicates are combined.

Table S1. List of guide RNAs used in this study

Experiment	description	name	Sequence (5'-3')	backbone
Sox2 tagging	KnockIn of eGFP/mCherry	Sox2_3'UTR	CAGCCCTCACATGTGCGACA	pX330
Landing pad's	KnockIn of HyTK at -116 kb	crRNA_Sox2_#23	TTGCCGGAACCTCTACCCGG	synthetic
	KnockIn of HyTK at -39 kb	crRNA_Sox2_#8	GCCACCTGATTGATACCTCG	synthetic
SCR deletion	SCR_deletion_5'	SCR_5'SCR_gRNA	GACGCTTCCGTTCTTGGAGT	pX330
	SCR_deletion_3'	SCR_3'SCR_gRNA	TTGGATTCCCGACAACAAGC	pX330
Sox2 deletion	upstream_of_Sox2P	up1c	TAGTTCTTGGGAACCCGGAA	pX330
	upstream of CTCF (Sox2P)	up2b	TGATTGTCCGCGCGCCACGA	pX330
	upstream of CTCF (Sox2P)	up2c	ATAAGTGGGAGGTTAAGCGA	pX330
	downstream of Sox2	do1a	ATGGCCGAATGATTAATAAC	pX330
	downstream of Sox2	do1b	TCGGCTCTGTTATTGGAATC	pX330
	downstream of Sox2	do1c	TGGCCGAATGATTAATAACG	pX330
Cutting control	cutting control Tdgf1	Tdgf1	AGGAACGCCCGGGTCTGTCA	pX458

Table S2. List of primers used in this study.

Experiment	Primer	Description	Sequence (5'-3')
genotyping *Sox2::mCherry/eGFP*	qME09	Sox2_Fw	CATGGGCTCTGTGGTCAAGT
	MEP232	Sox2_3'UTR_Rv	TCGGCAGCCTGATTCCAATA
	MEP68	Sox2_seqRv	CTGTCGTTTCGCTGCGGAGA
	EMp324	mCherry_Fw	CGTGGAACAGTACGAACGCG
	EMp325	eGFP_Fw	GTCCTGCTGGAGTTCGTGAC
SB Tagmentation	MEP009	SB_rev_enrich	tcctaactgacttgccaaaact
	LD027	SB_fwd_enrich	gtggtgatcctaactgacctaagac
	MEP011	SB_rev_pcr1	GTCTCGTGGGCTCGGAGATGTGTATAAGAGACAGaacgagttttaatgactccaactt
	MEP034	SB_fwd_pcr1	GTCTCGTGGGCTCGGAGATGTGTATAAGAGACAGggtgatcctaactgacctaagac
	MEP142	MEP142_PCR2	AATGATACGGCGACCACCGA
genotyping Sox2/ CBS_Sox2 deletions	CMP024	GFP_fw	catcaaggtgaacttcaagatccg
	CMP075	Sox2_del1_up_fw	GCCACATCTCAGAAACTAGGCG
	CMP077	Sox2_del2_up_fw	ATGCATAAACACCAGCCACCATTA
	CMP080	Sox2_del1_do_fw	CCAAATTAACGCAAAAACCGTGATG
	CMP081	Sox2_del1_do_rv	TGCAGAGATTTTCCGATTTGGGAC
genotyping pME034/ pME040/pME041	CMP111	Sox2_P_end_3_fw	CGTCGACgccaccatg
	CMP106	SV40_PAS_check_rv	actgcattctagttgtggtttgtcc
	CMP105	P2A_check_fw	tgaaacaagccggagatgtcg
genotyping LP -116	EM296	LP-116_Fw	ATGGGAGCATACCGATTCAC
	EM297	LP-116_Rv	CCCTGATTGTTTCCCGTAAT
	MEP105	MEP105_HyTK_Fw	tacccgagccgatgacttac
	oJOYC225	SB_Rv	aacgagttttaatgactccaactt
	oJOYC226	SB_Fw	Agtgagtttaaatgtatttggctaagg
genotyping LP -39	MEP173	gRNA_Sox2#8_locusFw	gataagatactgtgggcaaggg
	MEP174	gRNA_Sox2#8_locusRv	ggttatgtttgaggggatgtgt
	MEP178	gRNA_Sox2#8_locusFw 2	AGCTACAGTCCCACCCTCTA
	MEP179	gRNA_Sox2#8_locusRv 2	GCAGGCTTTGAGGTTTCGAA
genotyping LP -161	MEP206	LP-161kb_Fw	GGTCCCCGCTATTCAACTTAC
	MEP207	LP-161kb_Rv	GCGGTTTGCGGTCATTTAAA
cloning of loxP_CBS (pME012)	MEP74	FRT_donor	GAAGTTCCTATTCCGAAGTTCCTATTCTCTAGAAAGTATAGGAACTTCGCTAGCgtacCCTCGCG
	MEP75	F3_donor	GAAGTTCCTATTCCGAAGTTCCTATTCTTCAAATAGTATAGGAACTTCttagtatttggtagcatACTAGTgatcgaattATAACTTCGT
cloning of Sox2P-mTurq (pME034)	MEP145	Sox2_promoter_Fw	GTTCCCAAGAACTAAAGCAACCAAAACTTAAGGAGAAC
	MEP150	Sox2_prom_SalI_REV	GGTGGCGTCGACGCGGGCGCTGGGCGG
	MEP177	pME025_F3	GAAGTTCCTATTCCGAAGTTCCTATTCTTCAAATAGTATAGGAACTTCttgttaacttgtttattgcagcttataatggttac

	MEP147	Sox2_promoter_FRT_Fw	GAAGTTCCTATTCCGAAGTTCCTATTCTCTAGAAAGTATAGGAACTTCGTTCCCAAGAACTAAAGCAACCAAAACTTAAGGAGAAC
	MEP171	Sox2_prom_mT_polyA_Fw	gacgagctgtacaagTAATatccagacatgataagatacattgatgagtttgg
	MEP172	Sox2_prom_mT_polyA_Rv	GGCGAATTGGGCCCTcataaaatgaatgcaattgttgttgttaacttgtttattgc
cloning of Sox2P_CDS_mTurq (pME040 & pME041)	MEP222	CDS_SalI_Fw	CCAGCGCCCGCGTCGACATGTATAACATGATGGAGACG
	MEP223	Sox2_CDS_linker_GA_Rv	tttgttgctccggatccCATGTGCGACAGGGGCAG
	MEP224	mTurq_P2A_linker_GA_Fw	gatccggagcaacaaacttctctctgctgaaacaagccggagatgtcgaagagaatcctggaccggtgagcaagggcgaggag
	MEP225	mTurq_Rv_UTR_GA	TTCGCAGTCCAGCCCTTActtgtacagctcgtccatgcc
	MEP226	UTR_Fw	GGGCTGGACTGCGAACTG
	MEP230	UTR_F3_GA_RV_100nt	CAGTGTGATGGATGAAGTTCCTATTCCGAAGTTCCTATTCTTCAAATAGTATAGGAACTTCTTTCAGTGTCCATATTTCAAAAATTTATTTATCTCAAAC
	MEP231	mTurq_polyA_Rv	GCCGCCAGTGTGATGGATAT
cloning of new CDS-containing reporters (pCM038-pCM045)	CMP122	mTurq_fw_ov	CGCCCGCCCAGCGCCCGCGTCGACgccaccatggtgagcaagggcg
	CMP123	mTurq_SbfI_ov_rv	catcaatgtatcttaCCTGCAGGTTActtgtacagctcgtccatgccgag
	CMP124	SbfI_PAS_fw_ov	acaagTAACCTGCAGGtaagatacattgatgagtttggacaaacc
	CMP125	CDS_P2A_fw_ov	gcatggacgagctgtacaagggatccggagcaacaaacttctctctgctgaaacaagccggagatgtcgaagagaatcctggaccgTATAACATGATGGAGACGGAGCTGAAG
	CMP126	CDS_stop_rv_ov	aactcatcaatgtatcttaCCTGCAGGTTACATGTGCGACAGGGGCAG
	CMP127	stop_CDS_fw_ov	ctcggcatggacgagctgtacaagtaagTATAACATGATGGAGACGGAGCTGAAG
	CMP128	PAS_rv_ov	tccgtctccatcatgttatattgttaacttgtttattgcagcttataatggttac
	CMP129	Sox2CDS_fw	TATAACATGATGGAGACGGAGCTGAAG
	CMP130	Sox2CDS_F3_ov_rv	CCGCCAGTGTGATGGATATCTGCAGAATTCAGGGAAGTTCCTATTCCGAAGTTCCTATTCTTCAAATAGTATAGGAACTTCTTACATGTGCGACAGGGGC
	CMP131	revSox2_CDS_fw_ov	tggacgagctgtacaagTAACATGTGCGACAGGGGCAG
	CMP132	revSox2_CDS_rv_ov	actcatcaatgtatcttaCCTGCAGGATGTATAACATGATGGAGACGGAGCTG
	CMP133	mTurq_SbfI_rv_ovUTR	TTCGCAGTCCAGCCCCCTGCAGGTTActtgtacagctcgtccatgccgag
	CMP134	SbfI_3UTR_fw	CCTGCAGGGGGCTGGACTGCGAACTG
	CMP135	UTR_F3_rv	CGGCCGCCAGTGTGATGGAT
cloning of minimal sequence (pME037_min.seq_1561)	MEP190	MEP190_minSeq_Fw	AGAATACTCAAGCTATGCATCAAGCTTGGTACCGAGCTCGGAAGTTCCTATTCCGAAGTTCCTATTCTCTAGA
	MEP191	MEP191_minSeq_Rv	CGACTCACTATAGGGCGAATTGGGCCCTCTAGATGCATGCGAAGTTCCTATTCCGAAGTTCCTATTCTTCAAATAG
confirming hopping-induced deletions	CMP116	hopdel_allele_SNP2_fw	GGTATGTGGCATGATGATAGAGC
	CMP117	hopdel_allele_SNP2_rv	TGTTAAAGTGGCGTTATTGGC
	CMP118	hopdel_allele_SNP3_fw	GAGTAGGCAGTGAAAACATGACA
	CMP119	hopdel_allele_SNP3_rv	GGCAGCTTAATGAGTGAGGA

	CMP120	hopdel_allele_SNP4_fw	TCCTCCCATGATACAGCCAC
	CMP121	hopdel_allele_SNP4_rv	CCTTTCTACTCAGGCCGCTA

Table S3. List of plasmids used in this study

Plasmid name	Description	Addgene number (if available)
pME07	pME07_hPGK-SB100-IRES-NGFR-polyA	X
pME013	pME013_lb02_Fw_pZero	X
pME015	pME015_SB-FRT-mPGK-HyTK-F3-SB	X
pME024	pME024_HA-SB-HyTK-SB-HA	X
pME034	pME034_FRT_Sox2Prom_mTurq2_polyA_F3_pZero	X
pME037	pME037_pZero_min.seq_1561	X
pME040	pME040_Sox2P_CDS_mTurq_UTR	X
pME041	pME041_Sox2P_CDS_mTurq_polyA	X
pLD042	pLD042_hPGK-PB-IRES-NGFR-polyA	X
pCM038	pCM038_Sox2P_mTurq_P2A_CDS_PAS	X
pCM039	pCM039_Sox2P_mTurq_stop_CDS_PAS	X
pCM040	pCM040_Sox2P_mTurq_PAS_CDS	X
pCM041	pCM041_Sox2P_mTurq_revCDS_PAS	X
pCM043	pCM043_Sox2P_mTurq_P2A_CDS_UTR	X
pCM044	pCM044_Sox2P_mTurq_stop_CDS_UTR	X
pCM045	pCM045_Sox2P_mTurq_UTR_CDS	X
pJK14	pJK14 946 cre puro	X
pCAG-Flpe-addgene	pCAG-Flpe_addgene-plasmid-13787-sequence-7769	13787
HyTK-addgene	RV-L3-HyTK-2L_addgene-plasmid-11684-sequence-239457	11684
pX330-addgene	pX330-gRNA	158973
pCR-Zero	pCR Zero, #120275, Addgene	120275
pX458-addgene	pSpCas9(BB)-2A-GFP	48138
pLenti_mTurq2	pLenti-PGK-Puro-TK-NLS-mTurq2-PCNA	118617
pX330_Sox2	pX330-NQL005-SOX2-sgRNA	175553

Table S4. List of qPCR primers used in this study

Target	Primer	Description	Sequence (5'-3')
EGFP	SM67	GFP_Fw_qPCR	TCGTGACCACCCTGACCTAC
	SM68	GFP_Rv_qPCR	GGACTTGAAGAAGTCGTGCTGC
mTurquoise2	ME49	mTurq_2_Fw_qPCR	actttagcgacaacgtctatatcacc
	ME50	mTurq_2_Rv_qPCR	ttggggtctttgctcagctt
mCherry	SM72	mCherry_Fw_STE_qPCR_2	aggacggcgagttcatcta
	SM73	mCherry_Rv_STE_qPCR_2	cccatggtcttcttctgcatta
Gapdh	ME41	GAPDH_3_Fw_qPCR	CATCACTGCCACCCAGAAGACTG
	ME42	GAPDH_3_Rv_qPCR	ATGCCAGTGAGCTTCCCGTTCAG
Actin-B	ME43	Actb_Fw_qPCR	GGCTGTATTCCCCTCCATCG
	ME44	Actb_Rv_qPCR	CCAGTTGGTAACAATGCCATGT
Sox2	ME45	Sox2_2_Fw_qPCR	TACAGCATGTCCTACTCGCAG
	ME46	Sox2_2_Rv_qPCR	GAGGAAGAGGTAACCACGGG

Table S5. Sequences used as minimal insert and for proof-of-principle experiment

Description	Sequence (5'-3')
Arbitrary sequence insert for proof-of-principle experiment	GCTAGCgtacCCTCGCGGAGCCTTATGCCATACT CGTCCGCGGAGCACTCTGGTAATGCTTATGGT CCATAGGACATTCATCGCTTCCGGGTATGCGCT CTATTTGACCGTCTTTTGGCGCACAAATGCTGA CCACGAATTAAATTAGAGCGACTGCACAACTG TAAGGTCCGTCACGCAGACGACAGCCCAGGGA GACCACTGACCCATaagcttATAACTTCGTATAGC ATACATTATACGAAGTTATaattcgatcACTAGTatgc taccaaatactaa
Minimal insert	GAAGTTCCTATTCCGAAGTTCCTATTCTCTAGA AAGTATAGGAACTTCTGCACGGTATATTAAAC GTAGCGTCTCGAAGACTAGCAGAGCAATGGAG AGAAGTTCCTATACTATTTGAAGAATAGGAAC TTCGGAATAGGAACTTC

Table S6. List of external datasets used in this study

Data type	Cell type	Target	Source	ID	Used in Figure
RCMC	mESCs	Sox2 region	(*18*)	GSM6281849	Fig.1B, 2I, S6B
ATAC	mESCs		(*20*)	GSE98390	Fig. 2I, S4G
ChIP-seq	mESCs	H3K27ac	(*21*)	ENCFF583WVZ	Fig. 2I, S4G
ChIP-seq	mESCs	CTCF	Own data	GSE274894	Fig. 2I, S4G
ChIP-seq	mESCs	H3K27me3	(*57*)	ENCFF595SIA/ GSE208495	Fig. S4G
ChIP-seq	mESCs	Ring1B	(*17*)	GSE96107	Fig. S4G
ChIP-seq	mESCs	E2F1	(*58*)	GSM288349	Fig S6J
ChIP-seq	mESCs	POU5F1	(*59*)	GSM1587027	Fig S6J
ChIP-seq	mESCs	RXRA	(*60*)	GSM1603269	Fig S6J
ChIP-seq	mESCs	YY1	(*61*)	GSM788496	Fig S6J
ChIP-seq	mESCs	ZFP57	(*62*)	GSM1335222	Fig S6J
ChIP-seq	mESCs	ZIC2	(*63*)	GSM1499116	Fig S6J

Table S7. Number of mapped integrations per population

Number of mapped integrations per population per genomic region and the percentage of the integrations of that population genome-wide. The 2Mb region is the 2Mb around the *Sox2*-SCR locus (chr3:33643960-35643960) as in **Fig. 2C**, '*Sox2*-SCR region' is the 240kb region in **Fig. 2D** (chr3: 34590000-34830000).

**Cell line**	**population**	**integrations genome-wide**	**integrations in 2Mb region (% of genome-wide)**	**integrations in *Sox2*-SCR region (% of genome-wide)**
CRE6 -116 kb (proof-of-principle experiment)	ctrl	4865	2536 (52%)	876 (18%)
Sox2P-reporter -161 kb	*all*	*5961*	*2270 (38%)*	*1784 (30%)*
Sox2P-reporter -161 kb	ctrl	3536	624 (18%)	283 (8%)
Sox2P-reporter -161 kb	P1	8	8 (100%)	8 (100%)
Sox2P-reporter -161 kb	P2	520	428 (82%)	421 (81%)
Sox2P-reporter -161 kb	P3	662	498 (75%)	488 (74%)
Sox2P-reporter -161 kb	P4	568	365 (64%)	352 (62%)
Sox2P-reporter -161 kb	P5	421	203 (48%)	143 (34%)
Sox2P-reporter -161 kb	P6	246	144 (59%)	89 (36%)
Sox2P-reporter -116 kb	*all*	*8303*	*3094 (37%)*	*1973 (24%)*
Sox2P-reporter -116 kb	ctrl	4943	1150 (23%)	533 (11%)
Sox2P-reporter -116 kb	P1	44	37 (84%)	36 (82%)
Sox2P-reporter -116 kb	P2	937	791 (84%)	759 (81%)
Sox2P-reporter -116 kb	P3	673	381 (57%)	277 (41%)
Sox2P-reporter -116 kb	P4	395	163 (41%)	97 (25%)
Sox2P-reporter -116 kb	P5	436	218 (50%)	141 (32%)
Sox2P-reporter -116 kb	P6	875	354 (40%)	130 (15%)
Sox2P-reporter +715 kb	*all*	*2182*	*1134 (52%)*	*488 (22%)*
Sox2P-reporter +715 kb	ctrl	1265	496 (39%)	63 (5%)
Sox2P-reporter +715 kb	P1	7	7 (100%)	5 (71%)
Sox2P-reporter +715 kb	P2	57	51 (89%)	46 (81%)
Sox2P-reporter +715 kb	P3	209	158 (76%)	145 (69%)
Sox2P-reporter +715 kb	P4	254	184 (72%)	170 (67%)
Sox2P-reporter +715 kb	P5	234	144 (62%)	51 (22%)
Sox2P-reporter +715 kb	P6	156	94 (60%)	8 (5%)
Sox2P-reporter -161 kb Sox2::mCherry deleted	*all*	*11044*	*4617 (42%)*	*2970 (27%)*
Sox2P-reporter -161 kb Sox2::mCherry deleted	ctrl	3520	596 (17%)	333 (9%)
Sox2P-reporter -161 kb Sox2::mCherry deleted	P1_H	264	248 (94%)	246 (93%)
Sox2P-reporter -161 kb Sox2::mCherry deleted	P1	1027	1008 (98%)	1007 (98%)
Sox2P-reporter -161 kb Sox2::mCherry deleted	P1_L	671	650 (97%)	642 (96%)
Sox2P-reporter -161 kb Sox2::mCherry deleted	P2_H	60	51 (85%)	44 (73%)
Sox2P-reporter -161 kb Sox2::mCherry deleted	P2	261	181 (69%)	158 (61%)
Sox2P-reporter -161 kb Sox2::mCherry deleted	P2_L	40	23 (58%)	16 (40%)
Sox2P-reporter -161 kb Sox2::mCherry deleted	P3	223	123 (55%)	88 (39%)
Sox2P-reporter -161 kb Sox2::mCherry deleted	P4	255	156 (61%)	112 (44%)
Sox2P-reporter -161 kb Sox2::mCherry deleted	P5	594	422 (71%)	222 (37%)
Sox2P-reporter -161 kb Sox2::mCherry deleted	P6	4129	1159 (28%)	102 (2%)
Sox2P_CDS-reporter -161 kb	*all*	*6039*	*2908 (48%)*	*2445 (40%)*
Sox2P_CDS-reporter -161 kb	ctrl	3008	589 (20%)	283 (9%)
Sox2P_CDS-reporter -161 kb	P2	739	658 (89%)	655 (89%)
Sox2P_CDS-reporter -161 kb	P3	904	777 (86%)	768 (85%)
Sox2P_CDS-reporter -161 kb	P4	988	651 (66%)	617 (62%)
Sox2P_CDS-reporter -161 kb	P5	214	127 (59%)	67 (31%)
Sox2P_CDS-reporter -161 kb	P6	186	106 (57%)	55 (30%)
